# Effectiveness of Digital Mental Health Tools to Reduce Depressive and Anxiety Symptoms in Low- and Middle-Income Countries: Systematic Review and Meta-analysis

**DOI:** 10.2196/43066

**Published:** 2023-03-20

**Authors:** Jiyeong Kim, Lois M D Aryee, Heejung Bang, Steffi Prajogo, Yong K Choi, Jeffrey S Hoch, Elizabeth L Prado

**Affiliations:** 1 Department of Public Health Sciences School of Medicine University of California, Davis Davis, CA United States; 2 Department of Nutrition and Food Science University of Ghana Accra Ghana; 3 Division of Biostatistics, Department of Public Health Sciences School of Medicine, University of California, Davis Davis, CA United States; 4 Johns Hopkins Bayview Medical Center Baltimore, MD United States; 5 Department of Health Information Management School of Health and Rehabilitation Sciences, University of Pittsburgh Pittsburgh, PA United States; 6 Division of Health Policy and Management, Department of Public Health Sciences School of Medicine, University of California, Davis Davis, CA United States; 7 Department of Nutrition, Institute for Global Nutrition University of California, Davis Davis, CA United States

**Keywords:** digital mental health, mHealth, mobile health, digital health, low- and middle-income country, depression, anxiety, mobile phone

## Abstract

**Background:**

Depression and anxiety contribute to an estimated 74.6 million years of life with disability, and 80% of this burden occurs in low- and middle-income countries (LMICs), where there is a large gap in care.

**Objective:**

We aimed to systematically synthesize available evidence and quantify the effectiveness of digital mental health interventions in reducing depression and anxiety in LMICs.

**Methods:**

In this systematic review and meta-analysis, we searched PubMed, Embase, and Cochrane databases from the inception date to February 2022. We included randomized controlled trials conducted in LMICs that compared groups that received digital health interventions with controls (active control, treatment as usual, or no intervention) on depression or anxiety symptoms. Two reviewers independently extracted summary data reported in the papers and performed study quality assessments. The outcomes were postintervention measures of depression or anxiety symptoms (Hedges *g*). We calculated the pooled effect size weighted by inverse variance.

**Results:**

Among 11,196 retrieved records, we included 80 studies in the meta-analysis (12,070 participants n=6052, 50.14% in the intervention group and n=6018, 49.85% in the control group) and 96 studies in the systematic review. The pooled effect sizes were −0.61 (95% CI −0.78 to −0.44; n=67 comparisons) for depression and −0.73 (95% CI −0.93 to −0.53; n=65 comparisons) for anxiety, indicating that digital health intervention groups had lower postintervention depression and anxiety symptoms compared with controls. Although heterogeneity was considerable (*I*^2^=0.94 for depression and 0.95 for anxiety), we found notable sources of variability between the studies, including intervention content, depression or anxiety symptom severity, control type, and age. Grading of Recommendations, Assessments, Development, and Evaluation showed that the evidence quality was overall high.

**Conclusions:**

Digital mental health tools are moderately to highly effective in reducing depression and anxiety symptoms in LMICs. Thus, they could be effective options to close the gap in depression and anxiety care in LMICs, where the usual mental health care is minimal.

**Trial Registration:**

PROSPERO CRD42021289709; https://www.crd.york.ac.uk/prospero/display_record.php?RecordID=289709

## Introduction

### Background

Depressive and anxiety disorders are a leading cause of the global burden of disease [1]. Depression and anxiety contribute to an estimated 74.6 million years lived with disability, and 80% of this burden occurs in low- and middle-income countries (LMICs) [2]. However, the investment in mental health disorder prevention and treatment is substantially lower in LMICs than in high-income countries (HICs; $2 vs $50 per person in HICs) [3]. In LMICs, 80% to 95% of people with depression and anxiety do not receive the necessary mental health care, mainly because of the limited availability of service providers [4,5]. This large treatment gap could lead to detrimental consequences for the overall health of individuals with mental disorders, social and economic burden on their families, and large-scale societal loss in terms of decreased economic productivity owing to missed work (absenteeism) or reduced efficiency at work (presenteeism) [4,6].

In recent decades, the use of digital mental health (DMH) services to deliver mental health care has been increasing through the internet and other forms of technologies [7,8]. In this paper, we use the term DMH tools to refer to the following: either a digital platform as a tool to deliver mental health care (eg, cognitive behavioral therapy [CBT] provided by a service provider remotely via computer) or a digital platform itself as a main mental health intervention (eg, smartphone apps for depression). DMH tools have shown effectiveness among various populations in HICs, including youth, adults, older adults, and antenatal and postpartum women, in reducing conditions, such as mild to moderate or severe depression, social anxiety, panic disorder, suicidal ideation, posttraumatic stress disorder, attention-deficit/hyperactivity disorder, or insomnia [7,8]. A growing body of literature has started investigating barriers and facilitators for the successful implementation [9], usability, and acceptability to enhance user engagement with DMH tools [10,11]. Economic evaluations of DMH tools have reported that they are cost-effective [12,13]. These investigations informed us that well-designed DMH interventions could enhance access to quality mental health care with low-cost investment, which is ideal for LMICs. Indeed, the World Health Organization recognized the potential role of technology-supported mental health care tools in closing mental health treatment gaps in LMICs [14]. As the technology-enabling environment is expanding in LMICs (eg, 90% mobile phone penetration rate and 40% average internet connectivity) [15-17], diverse DMH interventions have been tested [18].

### Prior Work

A few systematic reviews examined digital health interventions for mental disorders in LMICs and reported several limitations [19], including that the quality of studies was suboptimal [20] and most studies reported short-term follow-ups and a low retention rate [21]. Thus, the findings were inconclusive in determining the clinical impact of DMH interventions. A recent meta-analysis reported a moderate effect of digital psychological interventions on mental disorders in LMICs [22]. However, the results were not explicit for depression or anxiety, which are the most prevalent forms of mental health issues [1]. Of note, this study was restricted to adult populations. This is a notable limitation given that (1) adolescents and children have escalating mental health care needs and (2) technology-enabled mental health care delivery is a promising strategy for these age groups [23,24]. Moreover, under the unprecedented SARS/COVID-19 pandemic, technology-based mental health interventions have surged, and overall mental distress was elevated in LMICs [25]. Hence, we need to investigate the effectiveness of DMH tools on depression and anxiety in acquiring comprehensive knowledge of this promising strategy in depression and anxiety care in low-resource settings. Therefore, this study aimed to quantify the effectiveness of digital health tools in reducing depression and anxiety in adults, adolescents, and children in LMICs.

## Methods

### Search Strategy and Study Selection

We searched the PubMed, Embase, and Cochrane databases for papers without language restrictions from database inception to February 22, 2022. We adapted our search strategy from previous reviews and settings [22,26] and refined it further to tailor it to our review purpose. Search terms included text, keywords, and Medical Subject Headings for PubMed/Medline and Cochrane databases, and the expansion (/exp) function was applied in Embase in the following three main areas: (1) technology; (2) mental health (depression or anxiety); and (3) LMICs, based on the World Bank Country Classification of the year the study was conducted. The full search terms for each database are provided in Multimedia Appendix 1.

During the full-text review, a snowball search was applied to find relevant studies from the references of previous systematic reviews. In addition, papers suggested by the citation program (Mendeley) were screened. We used the Google Translate software for data extraction and quality assessment of papers published in non-English languages. We also screened papers on ClinicalTrials.gov and the International Clinical Trials Registry Platform via the Cochrane Library to find unpublished clinical trials.

Studies were included if they (1) were randomized controlled trials (RCTs), (2) used technology (eg, computer, tablet PC, internet, mobile app, telephone, texting, or video or audio files) either as a tool to deliver traditional mental health care or the technology itself was a main mental health intervention, (3) measured depression or anxiety as either a primary or secondary outcome, (4) targeted people with low to moderate severity of depression or anxiety (eg, from lightly symptomatic to moderate disorder), (5) were conducted in an LMIC, and (6) used any comparison group (eg, active control, treat as usual, no intervention, or waitlist control).

Widely used standardized programs were used for abstract screening (Covidence) and full-text review (Microsoft Excel). Two reviewers (JK and SP) independently screened the abstracts. JK extracted and double-checked the data. SP independently extracted the data from 20% of randomly selected papers. If there were disagreements during screening and full-text review for inclusion, a third reviewer (HB) resolved the conflict. We applied the same inclusion and exclusion criteria to both the systematic review and the meta-analysis. The primary reviewer (JK) imported the extracted data into the summary table, and another reviewer (LMDA) checked the data in the summary table for accuracy. All the coauthors reviewed the data in the summary table. The biostatistician (HB) checked a random sample of the final data for effect size calculation.

### Data Analysis

The authors extracted the study population (demographics), study aims, settings, region, inclusion and exclusion criteria, interventions and controls (duration and details of procedures), outcome means, SDs, and measurement instruments. We contacted the corresponding authors to secure necessary data that were missing from published studies and excluded studies from the meta-analysis if the requested data were not provided. We evaluated an individual study’s methodological quality and risk of bias using the Effective Public Health Practice Project quality assessment tool in the following domains: selection bias, study design, confounders, blinding, data collection methods, withdrawal and dropout, intervention integrity, and analyses. Three reviewers assessed the listed domains independently (JK, LMDA, and SP), and the assessments were compared between the two assessors. Each domain was rated as 1=strong, 2=moderate, and 3=weak, and a global rating was assigned based on the section ratings as follows: 1=strong (no weak ratings), 2=moderate (1 weak rating), and 3=weak (2 or more weak ratings). In case of conflict, assessors discussed the discrepancies and reconciled the ratings. See Multimedia Appendix 2 [26-121] for our Effective Public Health Practice Project assessment. The quality of evidence for the outcome across all studies was assessed using the GRADE (Grading of Recommendations Assessment, Development, and Evaluation) criteria—risk of bias, inconsistency of effect, imprecision, indirectness, and publication bias (JK and LMDA). See Multimedia Appendix 3 for our GRADE assessment.

Stata 17 (StataCorp) was used to compute pooled effect sizes with 95% CIs. As the studies reported outcomes with different measurement tools, we calculated the Hedges *g* values with 95% CI as a standardized mean difference index to estimate the effect size. We chose Hedges *g* over Cohen *d*, because it is less prone to bias for the small sample studies, some of which were contained in our review [122]. Postintervention means and SDs were used for pooled effect size calculation. Weights were assigned to each study by calculating the inverse variance of the outcome scores. If more than one mental health measurement was reported, we included the one that was reported in the largest number of other studies. In the multiple arm studies, digital intervention was compared with each arm, and the effect size for each comparison was estimated. In this case, the frequency (N) of the control was divided by the number of comparisons to avoid overweighting the studies with multiple arms. We selected a random effect model to calculate pooled effect sizes, because we expected the studies to be dissimilar, whereas we wanted to generalize the results to other populations. Higgins and Thompson *I^2^* values were calculated to evaluate the heterogeneity across studies. We conducted subgroup analyses to investigate the variations in effect sizes by the following study characteristics: intervention content, type of technology use, mode, intervention duration, outcome measurement, depression or anxiety severity, control type, participant age, and study region. Furthermore, we performed a univariate, random-effect meta-regression using prespecified study characteristics (eg, study quality and blinding, in addition to the characteristics examined for the subgroup analysis) to look for notable sources of heterogeneity.

We conducted preplanned sensitivity analyses to test the robustness of the results for the studies where (1) the quality was poor, (2) more than one depression or anxiety score was reported, (3) the baseline psychometric scores were considerably different between the intervention and control groups, and (4) the digital tool was adjunct to the main nondigital intervention. We performed a post hoc sensitivity analysis, excluding outliers defined as the estimates’ 95% CI values that did not overlap with the 95% CI values of the pooled effect size [123]. To assess small-study effects and publication bias, we visually examined funnel plots and performed Egger weighted regression test to quantitatively evaluate the degree of asymmetry. To calculate the bias-corrected overall effect sizes after accounting for the publication bias and small-study effects, the Duval and Tweedie trim and fill method was used. The study’s review protocol was registered in PROSPERO (CRD42021289709), and the review process was compliant with the PRISMA (Preferred Reporting Item for Systematic Reviews and Meta-Analyses) guidelines, which can be found in Multimedia Appendix 4.

## Results

### Characteristics of the Included Studies

**Figure 1:**
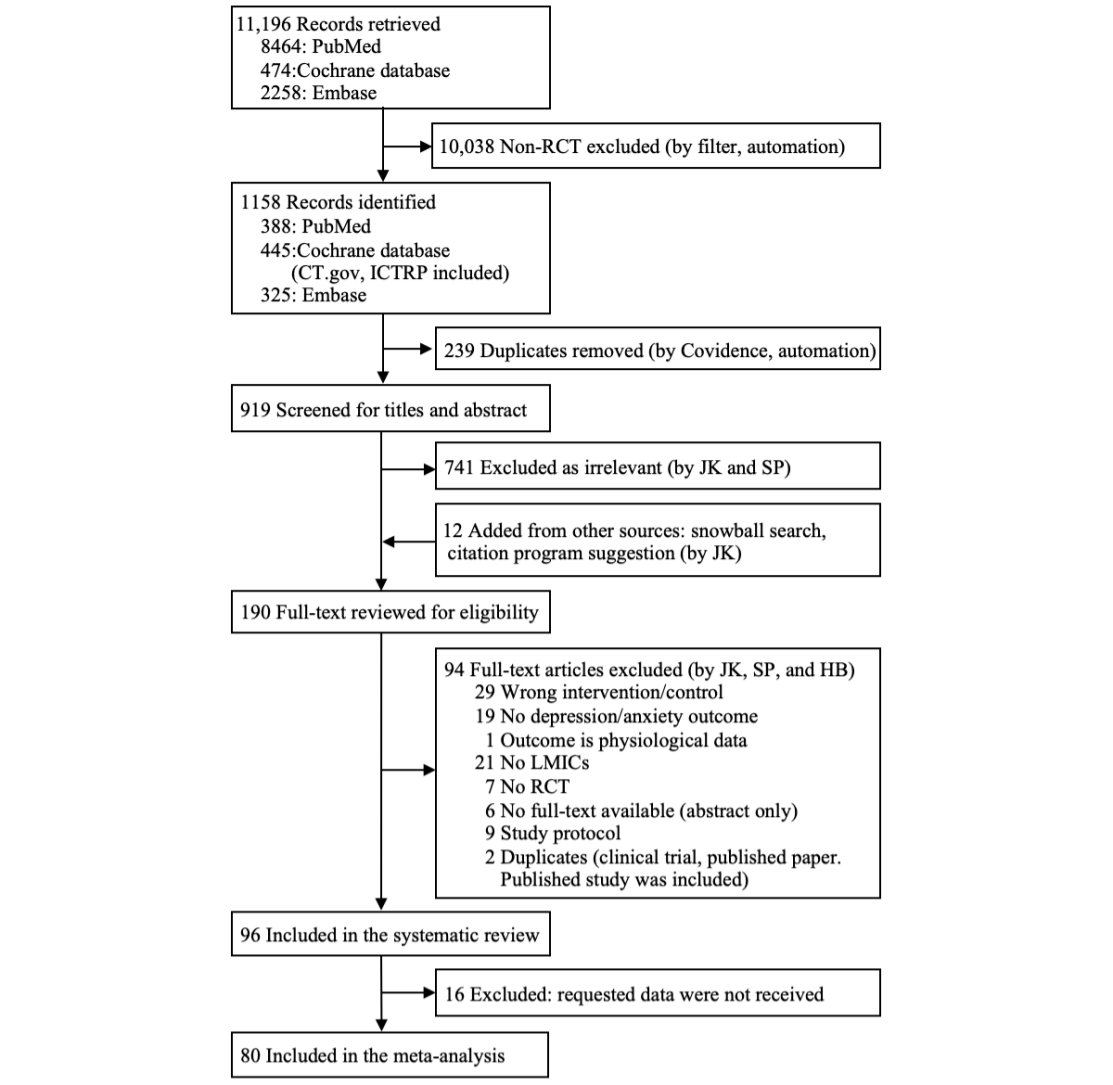
Study selection. CT.gov: ClinicalTrials.gov; ICTRP: International Clinical Trials Registry Platform; LMIC: low- and middle-income country; RCT: randomized controlled trial.

Initially, 11,196 records were retrieved and 1158 records were left after the RCT filter. When the records were imported to Covidence, 239 duplicates were removed automatically. We screened 919 studies for titles and abstracts and excluded 741 records as irrelevant. In addition, 12 studies were added from other sources; hence, a total of 190 studies were full-text reviewed and 94 studies were further excluded. We included 10 studies out of 22 from the recent meta-analysis, whereas the studies that were conducted in HICs and targeted posttraumatic stress disorder or substance abuse without depression or anxiety measurements were excluded [22]. A total of 96 studies were selected for the systematic review, and 80 studies were included for the meta-analysis, because data for 16 studies were not available to calculate the postintervention effect size (Figure 1).

Multimedia Appendix 5 [26-121] shows the characteristics of collected data from each study included in this systematic review. More information about the included studies is available in Multimedia Appendix 6 [26-121]. The meta-analysis contained a total of 12,070 participants (n=6052, 50.14% in the technology intervention group and n=6018, 49.85% in the control group). The participants’ age range was broad (4-75 years). The average number of participants per study was 144, ranging from 19 to 954. There were 59 and 54 studies reporting depression and anxiety outcomes, respectively. The measurement instruments were various, with 11 types for depression and 17 for anxiety. Symptoms were self-reported by the study participants. All 96 studies included in the systematic review were conducted between 2011 and 2021 in LMICs, including Asia (n=68), Africa (n=6), Europe (n=15), and Latin America (n=7). Studies published in Chinese and Portuguese were translated into English (n=3). In most studies (80/96, 83%), technology itself was a primary intervention, whereas technology was a tool to deliver intervention contents in 16 studies. Mobile apps and internet were the most common technology formats (80/96, 83%). Multiple contents (eg, psychotherapy plus peer support) were most frequently provided (33/96, 34%), followed by psychotherapy, including CBT (24/96, 25%). Playful distraction was primarily observed in relieving preprocedure anxiety (eg, dental treatment, venipuncture, and bone marrow aspiration), which was usually based on a single session and performed for children. The median intervention duration was 6 weeks, ranging from 1 day to 72 weeks. Some studies (13/96, 14%) had more than 2 study groups (eg, internet-based CBT vs in-person CBT vs no intervention). Usual in-person care or active control (65/96, 68%) was more common than no intervention as a control. Only 15% (14/96) of the studies provided adverse event information.

The study quality assessment results revealed that 80% (77/96) of the studies were considered moderate-to-high quality. The randomization method was described in most reports (72/96, 75%), and 91% (87/96) were considered likely to have representative sample populations. Hence, the possibility of selection bias seemed low. Approximately 77% (74/96) showed less than 20% attrition rates, and 65% (62/96) performed intention-to-treat analysis to account for the missing data. We rated the overall quality of evidence as high because we only included RCTs and did not downgrade it in the key 5 criteria—risk of bias, inconsistency, indirectness, imprecision, and publication bias. First, risk of bias was not considered high regarding randomization, blinding, attrition, and selective reporting. Second, outcomes were considered consistent because the study conclusions were consistent, and pooled effect sizes have narrow CIs. Although heterogeneity was considerable, the likely sources of heterogeneity were comprehensively suggested. Third, indirectness was low, as all the outcomes can directly answer our research question. Fourth, imprecision was considered low because the effect estimate was calculated from a large number of total participants (n=12,070) and was precise according to the GRADE guidelines. Finally, the possibility of publication bias was low to moderate.

### Outcomes of the Included Studies

**Figure 2:**
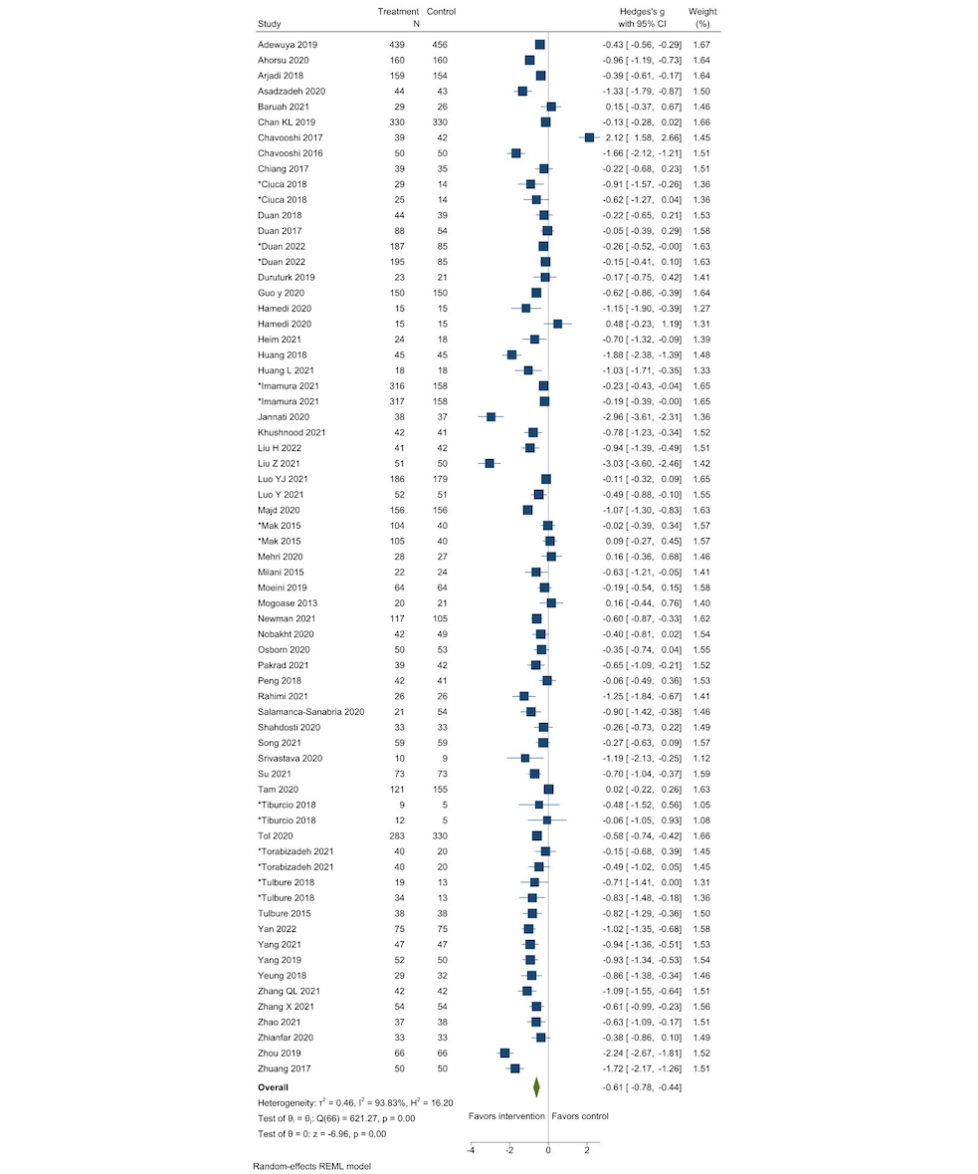
Effect of digital mental health interventions on depression. *Studies with more than 2 arms. The frequency (N) of the control group was divided by the number of comparisons to avoid being overweight. REML: restricted maximum likelihood.

**Figure 3:**
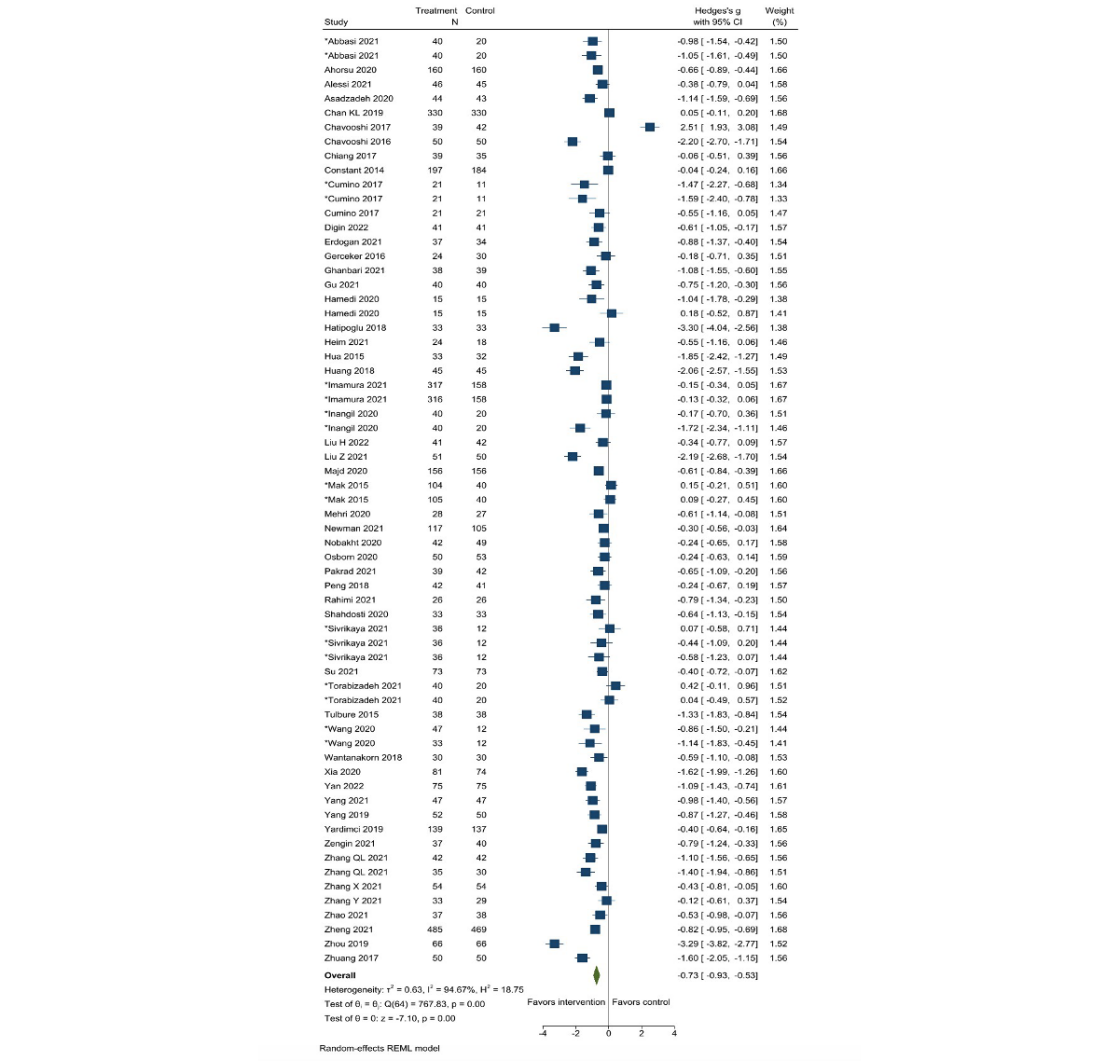
Effect of digital mental health interventions on anxiety. *Studies with more than 2 arms. The frequency (N) of the control group was divided by the number of comparisons to avoid being overweight. REML: restricted maximum likelihood.

We assessed the effects of DMH intervention compared with control groups with a mean (SD) from 80 studies (67 records for depression and 65 records for anxiety; Figure 2 [26, 28, 29, 32, 33, 35-39, 44-47, 54, 55, 57, 59-61, 63, 65, 68-78, 80, 82-84, 86-88, 90, 91, 93, 95, 97-101, 106, 107, 109, 111, 113, 115, 117, 119-121] and Figure 3 [27, 29, 30, 32, 35-38, 40, 42, 43, 48-50, 52, 55-59, 61, 62, 68, 69, 72-74, 78, 80, 82-84, 86, 88, 89, 93, 99, 101-103, 105-107, 109, 110, 112-118, 120, 121]). The pooled effect sizes were −0.61 (95% CI −0.78 to −0.44) for depression and −0.73 (95% CI −0.93 to −0.53) for anxiety, indicating that groups that used DMH tools had lower postintervention depression and anxiety symptoms compared with the controls. Considerable heterogeneity was observed (*I*^2^=0.94 for depression and 0.95 for anxiety). However, we were able to find the likely sources of heterogeneity, because the effect sizes were considerably different between prespecified subgroups (eg, by intervention content, technology type, mode, depression or anxiety level, outcome, and age) in both depression and anxiety (Table 1). For example, by intervention content, the largest effects on depression and anxiety were found among studies that provided CBT and other types of psychotherapy. By mode of delivery, internet- or mobile app–based interventions showed larger effect sizes compared with telephone- or text message–based approaches. By age, effect sizes on depression were larger among adults than among children, whereas effects on anxiety were larger among children than among adults. Although effect sizes were notably different among subgroups of studies, DMH interventions showed remarkably consistent pooled effects in reducing depression and anxiety symptoms among all subgroups of studies examined.

Notably, the effect size was larger (−0.70, 95% CI −0.90 to −0.51 for depression and −0.85, 95% CI −1.08 to −0.62 for anxiety) when we removed studies having considerable baseline score differences between groups in the sensitivity analysis. However, excluding outliers resulted in decreased effect sizes both in depression (−0.57, 95% CI −0.66 to −0.44) and anxiety (−0.66, 95% CI −0.77 to −0.56). Other sensitivity analyses did not change the effect sizes (Multimedia Appendix 7). Funnel plots appeared to be symmetrical for both anxiety and depression (Multimedia Appendix 8). Egger test results were not significant for depression (*P*=.19) but significant for anxiety (*P*=.03), suggesting potential publication bias for anxiety. However, when we ran the Duval and Tweedie trim and fill analysis to statistically assess the publication bias, no imputation was necessary to adjust for the publication bias in both depression and anxiety, and the effect sizes stayed the same.

**Table 1:** Subgroup analyses of digital mental health interventions.

Subgroup^a^		Depression				Anxiety			
		Frequency, n	Hedges *g* (95% CI)	*I*^2^ (%)	*P* value	Frequency, n	Hedges *g* (95% CI)	*I*^2^ (%)	*P* value
**By content**					<.001				<.001
	CBT^b^	18	−0.93 (−1.33 to −0.53)	94		10	−0.78 (−1.20 to −0.35)	94	
	Other psychotherapy	7	−0.58 (−1.64 to 0.48)	96		7	−0.82 (−2.15 to 0.52)	98	
	Psychoeducation	7	−0.30 (−0.45 to −0.16)	42		11	−0.27 (−0.61 to 0.07)	89	
	Multiple content	21	−0.62 (−0.85 to −0.39)	90		19	−0.75 (−0.98 to −0.53)	87	
	Physical activity	7	−0.29 (−0.47 to −0.11)	40		1	−0.53 (−0.98 to −0.07)	N/A^c^	
	Mindfulness	4	−0.36 (−0.84 to 0.11)	84		4	−0.26 (−0.73 to 0.21)	84	
	Social support	3	−0.59 (−1.02 to −0.15)	42		1	−0.64 (−1.13 to −0.16)	N/A	
	Playful distraction	N/A	N/A	N/A		12	−1.22 (−1.67 to −0.76)	86	
**By type of technology use**					<.001				.01
	Tech intervention	56	−0.58 (−0.73 to −0.44)	90		54	−0.74 (−0.94 to −0.55)	93	
	As a delivery tool	11	−0.74 (−1.52 to 0.05)	96		11	−0.65 (−1.41 to 0.11)	96	
**By mode**					.03				<.001
	Internet-based	37	−0.61 (−0.84 to −0.38)	92		19	−0.65 (−0.96 to −0.35)	91	
	Mobile apps	21	−0.75 (−0.50 to −1.00)	92		36	−0.92 (−1.18 to −0.66)	95	
	Telephone or Texting	8	−0.24 (−1.00 to −0.50)	96		9	−0.10 (−0.79 to 0.59)	96	
	Audio or video files	1	−0.58 (−0.74 to −0.42)	N/A		1	−1.05 (−1.61 to −0.49)	N/A	
**By duration**					.49				.005
	Multiple session	66	−0.62 (−0.79 to −0.44)	94		50	−0.71 (−0.96 to −0.46)	96	
	Single session	1	−0.35 (−0.74 to 0.04)	N/A		15	−0.80 (−1.06 to −0.53)	71	
**By depression or anxiety severity**					<.001				<.001
	Disorder	19	−0.91 (−1.25 to −0.56)	95		10	−0.96 (−1.30 to −0.62)	82	
	Symptomatic	48	−0.50 (−0.69 to −0.31)	93		55	−0.70 (−0.93 to −0.47)	95	
**By outcome**					<.001				.007
	Primary	60	−0.57 (−0.76 to −0.39)	94		58	−0.73 (−0.95 to −0.51)	94	
	Secondary	7	−0.94 (−1.24 to −0.65)	85		7	−0.73 (−1.27 to −0.19)	96	
**By control**					<.001				.91
	No intervention	22	−0.48 (−0.74 to −0.22)	89		22	−0.65 (−0.85 to −0.44)	73	
	Usual care or active	45	−0.67 (−0.90 to −0.45)	95		43	−0.77 (−1.06 to −0.48)	97	
**By age**					<.001				<.001
	Children	6	−0.32 (−0.76 to 0.12)	82		15	−1.05 (−1.46 to −0.63)	91	
	Adult	60	−0.63 (−0.82 to −0.45)	94		50	−0.64 (−0.87 to −0.41)	95	
	Older adult	1	−0.78 (−1.23 to −0.34)	N/A		N/A	N/A	N/A	
**By region**					.67				<.001
	Asia	53	−0.63 (−0.85 to −0.42)	95		46	−0.72 (−0.97 to −0.47)	96	
	Africa	3	−0.48 (−0.61 to −0.35)	29		2	−0.08 (−0.26 to 0.10)	0	
	Europe	8	−0.57 (−0.84 to −0.38)	35		13	−0.82 (−1.27 to −0.37)	91	
	Latin America	3	−0.62 (−1.14 to −0.10)	23		4	−0.93 (−1.54 to −0.32)	75	

^a^Intervention content (CBT, psychotherapy, psychoeducation, multiple contents, physical activity, mindfulness, social support, and playful distraction). Although CBT and mindfulness-based cognitive therapy are types of psychotherapy, we separated these from other types of psychotherapy. As CBT was the single most frequent, and mindfulness was emerging content for digital mental health tools, we intended to evaluate the effects separately: type of technology use (technology itself as an intervention or technology as a delivery tool); mode (internet-based, mobile apps, telephone or text messages, or audio or video files); intervention duration (multiple session or single session); depression or anxiety severity (depression or anxiety disorder or depression or anxiety at risk or simply symptomatic); outcome measurement (depression or anxiety is primary outcome or depression or anxiety is secondary outcome); control type (usual care or active control, no intervention, or waitlist control); participant age (children, adults, or older adults); and study region (Asia, Africa, Europe, or Latin America).

^b^CBT: cognitive behavioral therapy.

^c^N/A: not applicable.

## Discussion

### Principal Findings and Comparisons With Previous Work

With our 96 studies for systematic review and 80 studies for meta-analysis, we found that DMH interventions showed moderate to high effectiveness in reducing depression and anxiety symptoms in LMICs. Our findings contribute to knowledge-building in the effectiveness of DMH tools in LMICs, especially on depression and anxiety, which are the 2 most common mental disorders. The aggregated results from 80 RCTs with moderate to high effect sizes (Hedges *g*=−0.61 for depression and −0.73 for anxiety) provide a comprehensive up-to-date review (up to February 2022), with some promising evidence. Moreover, our findings are well aligned with previous systematic reviews in that DMH tools improved mental health outcomes in low-resource settings [20] with a moderate overall effect size (Hedges *g*=0.60) [22]. Our results are also comparable with the outcomes of digital psychological interventions in HICs that were found to be moderately effective in reducing depression (Hedges *g*=0.51-0.58) [124,125] and highly effective for anxiety (Hedges *g*=0.80) [126].

This study has some clinical implications for patient care. First, when psychotherapy, including CBT, was delivered via digital formats, it effectively relieved depression and anxiety symptoms compared with the usual care. Moreover, perinatal and postpartum depression was the single most frequent target for DMH intervention, followed by depression or anxiety care for the caregivers of children with chronic or congenital health conditions. Patients with chronic diseases were also often targeted. Thus, mobile app– or internet-based psychotherapy could be one way to care for the mental health of perinatal or postpartum women, patients with chronic disease, or their caregivers, who may be marginalized in mental health care in low-resource settings. Finally, our findings showed that digital tools (eg, social media and audiovisual materials) effectively alleviated preprocedure-related anxiety in the clinical setting.

### Limitations

Substantial heterogeneity should be acknowledged as a limitation of this study. It was anticipated because the main components of the study varied, including intervention formats, control types, and participants’ ages, which could lead to the large variance in effect sizes between studies. As *I*^2^ is an indicator of inconsistency across the study outcomes, high *I*^2^ signals that the observed variability could be real. We are also aware of the possibility of bias, including the small-study effect, because half of the studies had small sample sizes (eg, the total participants were <100). Small and pilot studies tend to include participants whose symptoms are likely more prominent or who are likely proactive and compliant with the study instructions. This could allow the studies to be well controlled and managed, potentially leading to a large overall effect size compared with what may occur in a real-world setting. Considering that DMH interventions tested through RCTs are still at an early stage in LMICs, it is not surprising that the sample size tends to be small, and the contents and study protocols vary substantially between studies. Moreover, the Egger test detected the possibility of publication bias (*P*=.03) for anxiety, although the trim and fill test revealed that no imputation was necessary for both outcomes to account for the publication bias. There is a possibility of duplication bias as we observed some studies conducted by the same research team, using similar study designs and interventions but for different target populations. We also need to recognize citation bias, as the notable findings are likely to be cited and included in our study. However, we believe that the possibility of language bias and availability or cost bias is minimal; we included non-English studies published in their local journals, and no papers were excluded owing to journal unavailability for full-text review. Finally, we were unable to adjust for the baseline scores, which could lead to the biased effect size or conservative results. For example, we witnessed some preintervention score differences between groups exceeding the postintervention score differences, meaning that the postintervention score differences could not correctly reflect the genuine effect of the intervention. In this study, this made the pooled effect size conservative for both depression and anxiety, resulting in increased effect sizes when these studies were excluded for sensitivity analysis.

### Study Strengths and Future Directions

This study has several strengths that can be acknowledged. First, our findings are aggregated results of 96 RCTs conducted in LMICs from 2011 to 2021. Hence, the moderate and high pooled effect sizes for depression and anxiety are supported by a large number of participants included. Second, our study focused on the most common mental disorders—depression and anxiety. The participants included those with both light depression or anxiety symptoms and moderate depression or anxiety disorders. The participants’ age range was broad, from preschool children to older adults, and study settings varied, including hospitals, schools, or fully web based. Thus, the diversity of study populations and treatment delivery settings may contribute to the generalizability of our results and qualitative and quantitative comparisons between various subgroups. Third, we were able to detect potential sources of variability, which could possibly be the intervention content, control type, baseline depression or anxiety symptom severity, and participant age. Notable effect size differences by subgroup signaled that the effectiveness of DMH tools could be more effective in reducing depressive or anxiety symptoms when a certain content was applied (eg, CBT) or it was administered for a certain level of baseline depression or anxiety (eg, depression disorder). Notably, our findings showed that using mobile apps could be more effective than non–mobile app–based DMH tools in relieving depressive and anxiety symptoms, although all these interpretations need caution. The evidence of the source of variability for both depression and anxiety is novel, as this information was not available in previous reviews. Fourth, noticeably, the effect size was higher when the DMH tools were compared with usual care or active control than no intervention for depression. This could highlight that DMH could be as effective as usual in-person care, not just better than nothing. Finally, studies conducted during the COVID-19 pandemic were included, showing that DMH tools could be useful to deal with depression and anxiety even when in-person social connections were reduced during an unprecedented worldwide infectious disease outbreak. Our findings suggest that DMH tools could be viable options when infectious diseases increase the risk of in-person–based care. This could be encouraging, as we are still in the extended COVID-19 pandemic era, and reportedly, the overall mental distress had been elevated nearly universally among all populations, especially in LMICs. For future studies, we would suggest that more effort would be needed for economic evaluation of DMH tools to gauge the feasibility of implementation and economic consequences and implications in LMICs.

### Conclusions

Our findings from a systematic review and meta-analysis of 96 RCTs showed that DMH tools could be an effective method to care for depression and anxiety in low-resource settings where usual care is minimally available or feasible despite the care necessity and urgency. This study provides ample evidence-based insight regarding future directions of DMH use for depression and anxiety in LMICs, given the anticipated increasing demand, development, and implementation of DMH tools.

## Appendix

Multimedia Appendix 1 Search strategy.

Multimedia Appendix 2 Study quality assessment (Effective Public Health Practice Project).

Multimedia Appendix 3 Overall quality of evidence (Grading of Recommendations, Assessment, and Evaluation).

Multimedia Appendix 4 PRISMA (Preferred Reporting Item for Systematic Reviews and Meta-Analyses) 2020 Checklist.

Multimedia Appendix 5 Characteristics of studies included in the systematic review.

Multimedia Appendix 6 Supplemental information of the studies for the systematic review.

Multimedia Appendix 7 Supplemental information on the sensitivity analysis.

Multimedia Appendix 8 Funnel plots for depression and anxiety.

## Abbreviations

CBT: cognitive behavioral therapy
DMH: digital mental health
GRADE: Grading of Recommendations Assessment, Development, and Evaluation
HIC: high-income country
LMIC: low- and middle-income country
PRISMA: Preferred Reporting Item for Systematic Reviews and Meta-Analyses
RCT: randomized controlled trial

## Data Availability

All data extracted from the reported papers for this systematic review and meta-analysis will be made available with publication on reasonable request to the corresponding author.
