# Supplementary material for: Effectiveness of Digital Mental Health Tools to Reduce Depressive and Anxiety Symptoms in Low- and Middle-Income Countries: Systematic Review and Meta-analysis
Source: JMIR Ment Health. 2023 Mar 20;10:e43066. doi: 10.2196/43066 (PMC10131603; doi:10.2196/43066)
Supplement: Multimedia Appendix 1 [file mental_v10i1e43066_app1.pdf]

Multimedia Appendix 1. Search strategy

1) Cochrane database

#1 (digital mental health):ti,ab,kw

#2 (mobile phone or cell phone or smart phone or telephone or computer* or web-based* or tablet*):ti,ab,kw

#3 (text message* or SMS* or e-mail or voice message* or audio message* or CATI* or IVR* or video game):ti,ab,kw

#4 MeSH descriptor: [Text Messaging] 1 tree(s) exploded

#5 MeSH descriptor: [Cell Phone] explode all trees

#6 MeSH descriptor: [Mobile Applications] this term only

#7 MeSH descriptor: [Computers] this term only

#8 MeSH descriptor: [Internet-Based Intervention] this term only

#9 #1 or #2 or #3 or #4 or #5 or #6 or #7 or # 8

#10 (Afghanistan OR Benin OR Burkina Faso OR Burundi OR Cambodia OR Central African Republic OR Chad OR Comoros OR Congo OR Democratic Republic of Congo OR Eritrea OR Ethiopia OR The Gambia OR Guinea OR Guinea Bissau OR Haiti OR Democratic Republic of Korea OR Liberia OR Madagascar OR Malawi OR Mali OR Mozambique OR Nepal OR Niger OR Rwanda OR Sierra Leone OR Somalia OR South Sudan OR Tanzania OR Togo OR Uganda OR Zimbabwe OR Armenia OR Bangladesh OR Bhutan OR Bolivia OR Cabo Verde OR Cape Verde OR Cameroon OR Republic of Congo OR Cote d'Ivoire OR Djibouti OR Egypt OR El Salvador OR Georgia OR Ghana OR Guatemala OR Guyana OR Honduras OR India OR Indonesia OR Kenya OR Kiribati OR Kosovo OR Kyrgyz Republic OR Kyrgyzstan OR Lao PDR OR Laos OR Lesotho OR Mauritania OR Micronesia OR Moldova OR Morocco OR Myanmar OR Nicaragua OR Nigeria OR Pakistan OR Papua New Guinea OR Philippines OR Samoa OR Sao Tome and Principe OR Senegal OR Solomon Islands OR Sri Lanka OR Sudan OR Swaziland OR Syria OR Syrian Arab Republic OR Tajikistan OR Timor Leste OR Ukraine OR Uzbekistan OR Vanuatu OR Vietnam OR Yemen OR Zambia OR Albania OR Algeria OR American Samoa OR Angola OR Azerbaijan OR Belarus OR Belize OR Bosnia and Herzegovina OR Botswana OR Brazil OR Bulgaria OR China OR Colombia OR Costa Rica OR Cuba OR Dominica OR Dominican Republic OR Ecuador OR Fiji OR Gabon OR Grenada OR Iran OR Iraq OR Jamaica OR Jordan OR Kazakhstan OR Libya OR Macedonia OR Malaysia OR Maldives OR Marshall Islands OR Mauritius OR Mexico OR Mongolia OR Montenegro OR Namibia OR Palau OR Panama OR Paraguay OR Peru OR Romania OR Serbia OR South Africa OR St. Lucia OR St. Vincent and the Grenadines OR Suriname OR Thailand OR Tonga OR Tunisia OR Turkey OR Turkmenistan OR Tuvalu):ti,ab,kw

#11 MeSH descriptor: [Developing Countries] this term only

#12 #10 and #11

#13 (RCT* or randomized clinical* or randomized controlled*):ti,ab,kw

#14 (depression* or anxiety*):ti,ab,kw

#15 #9 and #12 and #13 and #14

474 articles retrieved → RCT filter applied. → Among those, 445 studies were RCT as of 2/22/2022. Hence, 445 were included for abstract screening.

2) PubMed/Medline

((((("Afghanistan" OR "Benin" OR "Burkina Faso" OR "Burundi" OR "Cambodia" OR "Central African Republic" OR "Chad" OR "Comoros" OR "Congo" OR "Democratic Republic of Congo" OR "Eritrea" OR "Ethiopia" OR "The Gambia" OR "Guinea" OR "GuineaBissau" OR "Haiti" OR "Democratic Republic of Korea" OR "Liberia" OR "Madagascar" OR "Malawi" OR "Mali" OR "Mozambique" OR "Nepal" OR "Niger" OR "Rwanda" OR "Sierra Leone" OR "Somalia" OR "South Sudan" OR "Tanzania" OR "Togo" OR "Uganda" OR "Zimbabwe")) OR ("Armenia" OR "Bangladesh" OR "Bhutan" OR "Bolivia" OR "Cabo Verde" OR "Cape Verde" OR "Cameroon" OR "Republic of Congo" OR "Cote d'Ivoire" OR "Djibouti" OR "Egypt" OR "El Salvador" OR "Georgia" OR "Ghana" OR "Guatemala" OR"Guyana" OR "Honduras" OR "India" OR "Indonesia" OR "Kenya" OR "Kiribati" OR "Kosovo" OR "Kyrgyz Republic" OR "Kyrgyzstan" OR "Lao PDR" OR "Laos" OR "Lesotho" OR "Mauritania" OR "Micronesia" OR "Moldova" OR "Morocco" OR "Myanmar" OR "Nicaragua" OR "Nigeria" OR "Pakistan" OR "Papua New Guinea" OR "Philippines"OR "Samoa" OR "Sao Tome and Principe" OR "Senegal" OR "Solomon Islands" OR "Sri Lanka" OR "Sudan" OR "Swaziland" OR "Syria" OR "Syrian Arab Republic" OR "Tajikistan" OR "TimorLeste" OR "Ukraine" OR "Uzbekistan" OR "Vanuatu" OR "Vietnam" OR "Yemen" OR "Zambia")) OR ("Albania" OR "Algeria" OR "American Samoa" OR "Angola" OR "Azerbaijan" OR "Belarus" OR "Belize" OR "Bosnia and Herzegovina" OR "Botswana" OR "Brazil" OR "Bulgaria" OR "China" OR "Colombia" OR "Costa Rica" OR "Cuba" OR "Dominica" OR "Dominican Republic" OR "Ecuador" OR "Fiji" OR "Gabon" OR "Grenada" OR "Iran" OR "Iraq" OR "Jamaica" OR "Jordan" OR "Kazakhstan" OR "Libya" OR "Macedonia" OR "Malaysia" OR "Maldives" OR "Marshall Islands" OR "Mauritius" OR "Mexico" OR "Mongolia" OR "Montenegro" OR "Namibia" OR "Palau" OR "Panama" OR "Paraguay" OR "Peru" OR "Romania" OR "Serbia" OR "South Africa" OR "St. Lucia" OR "St. Vincent and the Grenadines" OR "Suriname" OR "Thailand" OR "Tonga" OR "Tunisia" OR "Turkey" OR "Turkmenistan" OR "Tuvalu")) OR ("Developing Countries")[Mesh] OR ("Africa")[Mesh] OR ("Asia")[Mesh] OR ("South America")[Mesh] OR ("Caribbean Region")[Mesh] OR ("Central America")[Mesh])))
AND (((("Telemetry" OR "Telemedicine" OR "Telepsychiatry" OR "Telehealth" OR "Telecare" OR "Telemental health" OR "eHealth" OR "uHealth" OR "Connected Health" OR "online" OR "internet" OR "Internet health" OR "Web Browser" OR "Website" OR "Web-based" OR "Social Media" OR "Facebook" OR "Mobile Health" OR "Mobile Technology" OR "Mobile phone" OR "Cellular Phone" OR "Cellphone" OR "Smartphone" OR "text message" OR "SMS" OR "Artificial Intelligence" OR "Game Theory" OR "User Computer Interface" OR "Computer Simulation" OR "Speech Recognition Software" OR "Therapy, Computer Assisted" OR "Remote Sensing Technology" OR "Remote Consultation"))))

AND (((("psychotherapy" OR "serious mental illness" OR "serious and persistent mental illness" OR "severe mental illness" OR "mental illness" OR "mental health" OR "mental disorder" OR "major depressive disorder" OR "depression" OR "anxiety" OR "affective disorder" OR "posttraumatic stress disorder" OR "ptsd" OR "stress disorder" OR "antipsychotic"))))

8,464 retrieved → RCT filtered → 388 studies were included for abstract screening (2/22/2022)

3) Embase

#1 ‘mobile phone’/exp
#2 ‘text messaging’/exp
#3 ‘web-based intervention’/exp
#4 ‘mobile application’/exp
#5 ‘mobile phone’ or ‘cell phone’ or ‘smart phone’ or ‘smartphone’ or tablet’ or ‘computer-based’ or ‘internet-based’:ti,ab
#6 ‘digital mental health’:ti,ab
#7 ‘text messag$’ or ‘SMS’:ti,ab
#8 ‘social media’ or ‘website’ or ‘facebook’ or ‘twitter’ or ‘whats app’ or ‘email’:ti,ab
#9 ‘telepsychotherapy’ or ‘telemental$’ or ‘telehealth’ or ‘tele health’ or ‘e-health’ or tele behavior$’:ti,ab
#10 ‘depression’ or ‘anxiety’:ti,ab
#11 'Afghanistan' OR 'Benin' OR 'Burkina Faso' OR 'Burundi' OR 'Cambodia' OR 'Central African Republic' OR 'Chad' OR 'Comoros' OR 'Congo' OR 'Democratic Republic of Congo' OR 'Eritrea' OR 'Ethiopia' OR 'The Gambia' OR 'Guinea' OR 'Guinea Bissau' OR 'Haiti' OR 'Democratic Republic of Korea' OR 'Liberia' OR 'Madagascar' OR 'Malawi' OR 'Mali' OR 'Mozambique' OR 'Nepal' OR 'Niger' OR 'Rwanda' OR 'Sierra Leone' OR 'Somalia' OR 'South Sudan' OR 'Tanzania' OR 'Togo' OR 'Uganda' OR 'Zimbabwe' OR 'Armenia' OR 'Bangladesh' OR 'Bhutan' OR 'Bolivia' OR 'Cabo Verde' OR 'Cape Verde' OR 'Cameroon' OR 'Republic of Congo' OR 'Cote d’Ivoire' OR 'Djibouti' OR 'Egypt' OR 'El Salvador' OR 'Georgia' OR 'Ghana' OR 'Guatemala' OR 'Guyana' OR 'Honduras' OR 'India' OR 'Indonesia' OR 'Kenya' OR 'Kiribati' OR 'Kosovo' OR 'Kyrgyz Republic' OR 'Kyrgyzstan' OR 'Lao PDR' OR 'Laos' OR 'Lesotho' OR 'Mauritania' OR 'Micronesia' OR 'Moldova' OR 'Morocco' OR 'Myanmar' OR 'Nicaragua' OR 'Nigeria' OR 'Pakistan' OR 'Papua New Guinea' OR 'Philippines' OR 'Samoa' OR 'Sao Tome and Principe' OR 'Senegal' OR 'Solomon Islands' OR 'Sri Lanka' OR 'Sudan' OR 'Swaziland' OR 'Syria' OR 'Syrian Arab Republic' OR 'Tajikistan' OR 'Timor Leste' OR 'Ukraine' OR 'Uzbekistan' OR 'Vanuatu' OR 'Vietnam' OR 'Yemen' OR 'Zambia' OR 'Albania' OR 'Algeria' OR 'American Samoa' OR 'Angola' OR 'Azerbaijan' OR 'Belarus' OR 'Belize' OR 'Bosnia' and 'Herzegovina' OR 'Botswana' OR 'Brazil' OR 'Bulgaria' OR 'China' OR 'Colombia' OR 'Costa Rica' OR 'Cuba' OR 'Dominica' OR 'Dominican Republic' OR 'Ecuador' OR 'Fiji' OR 'Gabon' OR 'Grenada' OR 'Iran' OR 'Iraq' OR 'Jamaica' OR 'Jordan' OR 'Kazakhstan' OR 'Libya' OR 'Macedonia' OR 'Malaysia' OR 'Maldives' OR 'Marshall Islands' OR 'Mauritius' OR 'Mexico' OR 'Mongolia' OR 'Montenegro' OR 'Namibia' OR 'Palau' OR 'Panama' OR 'Paraguay' OR 'Peru' OR 'Romania' OR 'Serbia' OR 'South Africa' OR 'St. Lucia' OR 'St. Vincent and the Grenadines' OR 'Suriname' OR 'Thailand' OR 'Tonga' OR 'Tunisia' OR 'Turkey' OR 'Turkmenistan' OR 'Tuvalu':ti,ab
#12 ‘low-and middle-income countries’ or ‘lmic$’:ti,ab
#13 #1 or #2 or #3 or #4 or #5 or #6 or #7 or #8 or #9
#14 #11 or #12
#15 #10 and #13 and #14

2,258 retrieved → RCT filter applied → 325 studies were included for abstract screening (2/22/2022)
