# Supplementary material for: Effectiveness of Digital Mental Health Tools to Reduce Depressive and Anxiety Symptoms in Low- and Middle-Income Countries: Systematic Review and Meta-analysis
Source: JMIR Ment Health. 2023 Mar 20;10:e43066. doi: 10.2196/43066 (PMC10131603; doi:10.2196/43066)
Supplement: Multimedia Appendix 2 [file mental_v10i1e43066_app2.pdf]

Multimedia Appendix 2. Study Quality Assessment

1) Study quality assessment domains of the Effective Public Health Practice Project (EPHPP) quality assessment

Section A. Selection bias

A1. Are the individuals that were selected to participate in the study likely to be representative of the target population?
Participants are more likely to be representative of the target population if they are randomly selected from a comprehensive list of individuals in the target population (score very likely). They may not be representative if they are referred from a source (e.g., clinic) in a systematic manner (score somewhat likely) or self-referred (score not likely).

1.Very Likely
2.Somewhat Likely
3.Not Likely
4.Can't Tell

A2. What percentage of individuals selected for the study agreed to participate?
This refers to the % of subjects in the control and intervention groups that agreed to participate in the study before they were assigned to intervention or control groups.

**Usually, you can find this or calculate this from the study profile/flowchart/Figure 1.

1.80-100%
2.60-79%
3.Less than 60%
4.NA
5.Can't Tell

A Section Rating
How to rate this section:
1. Strong: The selected individuals are very likely to be representative of the target population (A1 is Very Likely) and there is greater than 80% participation (A2 is 80-100%).
2. Moderate: The selected individuals are at least somewhat likely to be representative of the target population (A1 is Very Likely or Somewhat Likely); and there is 60 - 79% participation (A2 is 60-79%). 'Moderate' may also be assigned if A1 is Very Likely or Somewhat Likely and A2 is 5 (can't tell).
3. Weak: The selected individuals are not likely to be representative of the target population (A1 is Not Likely); or there is less than 60% participation (A2 is Less than 60%) or selection is not described (A1 is Can't Tell); and the level of participation is not described (A2 is Not Described).

1.Strong
2.Moderate
3.Weak

Section B. Study design

B1. What is the study design?

1.Randomized Control Trial
2.Controlled Clinical Trial
3.Cohort analytic
(two group pre and post)
4.Case control study
5.Cohort (one group pre + post (before and after)
6.Interrupted time series
7.Other
8.Can't Tell

B2. Was the study described as randomized?
Score YES, if the authors used words such as random allocation, randomly assigned, and random assignment. Score NO, if no mention of randomization is made.

1. Yes
2. No

B3. If yes, was the method of randomization described?
YES, if the authors describe any method used to generate a random allocation sequence. Score NO, if the authors do not describe the allocation method or describe methods of allocation such as alternation, case record numbers, dates of birth, day of the week, and any allocation procedure that is entirely transparent before assignment, such as an open list of random numbers of assignments.

1.Yes
2.No

B4. If yes, was the method appropriate?
Score YES, if the randomization sequence allowed each study participant to have the same chance of receiving each intervention and the investigators could not predict which intervention was next. Examples of appropriate approaches include assignment of subjects by a central office unaware of subject characteristics, or sequentially numbered, sealed, opaque envelopes. Score NO, if the randomization sequence is open to the individuals responsible for recruiting and allocating participants or providing the intervention, since those individuals can influence the allocation process, either knowingly or unknowingly. If NO is scored, then the study is a controlled clinical trial.

1.Yes
2.No

B Section rating
How to rate this section:
1. Strong: will be assigned to those articles that described RCTs and CCTs.
2. Moderate: will be assigned to those that described a cohort analytic study, a case control study, a cohort design, or an interrupted time series.
3. Weak: will be assigned to those that used any other method or did not state the method used.

1. Strong
2.Moderate
3.Weak

Section C. Confounder

C1. Were there important differences between groups prior to the intervention?
The following are examples of confounders:
1. Race, 2. Sex, 3. Marital /family, 4. Age, 5. SES (income or class) , 6. Education, 7. Health status 8. Pre-intervention score on outcome measure

**Usually you can find this in Table 1, comparing baseline characteristics between groups. Look at what the authors say in the Results section about the baseline comparisons. If they say the groups were balanced, then score 2 (No). Even if the p-value for the difference between intervention groups is significant, the magnitude of the difference between groups might still be small so the difference might not be considered important. Or there might be a difference in only one of many baseline characteristics examined, which might also not be considered important. You might also find this stated in the Discussion section in a paragraph describing the strengths and weaknesses or limitations of the study. For example, the authors might say that a difference in baseline characteristics was a weakness of the study, in which case you would score 1 (yes). Or the authors might say that even though a significant difference in one of many baseline characteristics was found, the magnitude was small and this was not considered important (Score 2 - No).

1.Yes
2.No
3.Can't Tell

C2. If yes, indicate the percentage of relevant confounders that were controlled (either in the design (e.g., stratification, matching) or analysis)?
**It seems that "relevant confounders" are defined as any characteristics that were significantly or substantially different between groups at baseline. We will use this definition as the denominator to calculate the percentage of relevant confounders that were controlled or adjusted for in the analysis.

1.80-100%
2.60-79%
3.Less than 60%
4.Can't Tell

C Section rating
How to rate this section:
1. Strong: will be assigned to those articles that controlled for at least 80% of relevant confounders (C2 is 80-100%); or (C1 is No).
2. Moderate: will be given to those studies that controlled for 60 – 79% of relevant confounders (C1 is Yes) and (C2 is 60-79%).
3. Weak: will be assigned when less than 60% of relevant confounders were controlled (C2 is Less than 60%) and or control of confounders was not described (C1 is Can't Tell) and (C2 is Can't Tell).

1.Strong
2.Moderate
3.Weak

Section D. Blinding

D1. Was (were) the outcome assessor(s) aware of the intervention or exposure status of participants?
Were the individuals measuring the outcome aware of the intervention or group assignment?

Key words to search for:
- Blind
- Aware
- Know

1.Yes
2.No

D2. Were the study participants aware of their intervention or exposure status?
Key words to search for:
- Blind
- Aware
- Know

1.Yes
2.No

D Section rating
How to rate this section:
1. Strong: The outcome assessor is not aware of the intervention status of participants (D1 is No); and the study participants are not aware of their intervention status (D2 is No).
2. Moderate: The outcome assessor is not aware of the intervention status of participants (D1 is No); or the study participants are not aware of their intervention status (D2 is no); or blinding is not described (D1 is Not described and D2 is Not Described).
3. Weak: The outcome assessor is aware of the intervention status of participants (D1 is Yes); and the study participants are aware of their intervention status (D2 is Yes).

1. Strong
2.Moderate
3.Weak

Section E. Data collection method

E1. Were data collection tools shown to be valid?
Do the tools accurately measure what they are supposed to measure? This is usually assessed by comparing the score on the tool to a gold standard measure or can also look for associations with variables expected to be related to the score.

1.Yes
2.No

E2. Were data collection tools shown to be reliable?
Do the tools produce consistent results with repeated assessments? This is usually assessed by administering the tool to the same group of children twice and comparing the scores on the first and second assessment (rest/re-test reliability).

1.Yes
2.No

E Section rating
1. Strong: The data collection tools have been shown to be valid (E1 is Yes); and the data collection tools have been shown to be reliable (E2 is Yes).
2. Moderate: The data collection tools have been shown to be valid (E1 is Yes); and the data collection tools have not been shown to be reliable (E2 is No) or reliability is not described (E2 is Not Described).
3. Weak: The data collection tools have not been shown to be valid (E1 is No) or both reliability and validity are not described (E1 is Not Described and E2 is Not Described).

1. Strong
2. Moderate
3. Weak

Section F. Withdrawals and drop-outs

F1. Were withdrawals and drop-outs reported in terms of numbers and/or reasons per group?
Another term that is often used for drop-outs is "loss to follow-up." This means the same thing. Often times this will be reported in the start of the results section.

1.Yes
2.No

F2. Indicate the percentage of participants completing the study. (If the percentage differs by groups, record the lowest).
Keywords to search for:
- Loss
- Drop-out
- Attrition
- Study profile
- Figure 1

1. 80-100%
2.60-79%
3.Less than 60%
4.Can't Tell

F Section rating
How to rate this section:
1. Strong: will be assigned when the follow-up rate is 80% or greater (F2 is 80-100%).
2. Moderate: will be assigned when the follow-up rate is 60 – 79% (E2 is 60-79%) OR E2 is N/A.
3. Weak: will be assigned when a follow-up rate is less than 60% (E2 is Less than 60%) or if the withdrawals and drop-outs were not described (E2 is Not Described).

1.Strong
2.Moderate
3.Weak

Section G. Intervention integrity

G1. What percentage of participants received the allocated intervention or exposure of interest?
The numerator should be the number of study participants that received the intervention and the denominator should be the number of study participants in the intervention group).

In trials where the mother received an intervention during pregnancy and it was intended that her child would receive an intervention later as well, however, the mother was lost to follow-up prior to the child receiving the intervention, we will consider the child as having received the intervention. If this is the case, put a footnote in the spreadsheet explaining that this is what you did.

1.80-100%
2.60-79%
3.Less than 60%
4.Can't Tell

G2. Was the consistency of the intervention measured?
Did the investigators measure whether the intervention was delivered to participants in the same way every time?

1.Yes
2.No

G3. Is it likely that subjects received an unintended intervention (contamination or co-intervention) that may influence the results?
Contamination occurs when individuals outside of the intervention/treatment group (such as study participants in the control/comparison group or members of the community that are not enrolled in the study) are unintentionally exposed to the intervention in some way. Co-intervention occurs when study participants are exposed to another intervention outside of the study (such as an intervention from another research study or program), which may influence the outcomes of the study. If the authors do not say anything about likely contamination or co-intervention, then score "No." You do not need to make your own judgment about the likelihood of this occurring.

1.Yes
2.No

Section H. Analysis

H1. Indicate the unit of allocation (or randomization)? (1=individual, 2=cluster)
This should be stated straightforward in the text.

1.Individual
2.Cluster

H2. Indicate the unit of analysis? (1=individual, 2=cluster)
If individual level data were collected, unit of analysis is individual. This should be the case for all of our studies.

1. Individual
2. Cluster

H3. Are the statistical methods appropriate for the study design? (1=yes, 2=no, 3=can't tell)
In particular, if the unit of allocation was the cluster, did the investigators adjust for clustering?

1.Yes
2.No

H4. Is the analysis performed by intervention allocation status (i.e., intention to treat) rather than the actual intervention received?

Intention to treat means that study participants are analyzed according to the intervention and control/comparison groups to which they were originally assigned regardless of whether or not they received the intervention as intended. This can often be found in the statistical analysis section of the article.

1. Yes
2. No

Global ratings for the paper

1. STRONG: No WEAK ratings. Either all STRONG ratings; or 1-2 MODERATE ratings and the rest are STRONG.
2. MODERATE: More than 2 MODERATE ratings and the rest are STRONG; or 1 WEAK rating and the rest are STRONG.
3. WEAK: 2 or more WEAK ratings.

1. Strong
2. Moderate
3. Weak

Global quality rating of the included studies

AUTHOR	**Section A rating: Selection bias**<br>1.Strong<br>2.Moderate<br>3.Weak	**Section B rating: Study design**<br>1.Strong<br>2.Moderate<br>3.Weak	**Section C rating: Confounder**<br>1.Strong<br>2.Moderate<br>3.Weak	**Section D rating: Blinding**<br>1.Strong<br>2.Moderate<br>3.Weak	**Section E rating: Data collection methods**<br>1. Strong<br>2. Moderate<br>3. Weak	**Section F rating: Withdrawals and drop-outs**<br>1.Strong<br>2.Moderate<br>3.Weak	**Global rating of the study**<br>1.Strong<br>2.Moderate<br>3.Weak
Abbasi 2021	2	1	3	3	1	1	3
Adewuya 2019	1	1	1	1	1	2	1
Ahorsu 2020	2	1	1	1	1	1	1
Alessi 2021	1	1	1	3	1	1	2
Araya 2021	2	1	1	2	1	1	1
Arjadi 2018	3	1	1	1	1	1	2
Asadzadeh 2020	2	1	1	2	1	1	1
Baruah 2021	3	1	1	1	1	2	2
Byonanebye 2021	2	1	1	3	1	1	2
Chan KL 2019	2	1	1	2	1	2	2
Chavooshi 2017	3	1	1	2	1	1	2
Chavooshi 2016	2	1	1	1	1	2	2
Chiang 2017	1	1	1	1	1	1	1
Ciuca 2018	3	1	1	1	1	1	2

Constant 2014	2	1	1	2	1	1	1
Craveiro 2020	2	1	3	2	1	1	3
Cumino 2017	2	1	1	3	1	1	2
Digin 2022	2	1	1	3	1	1	2
Duan 2018	2	1	1	3	1	2	3
Duan 2017	2	1	3	2	1	2	3
Duan 2022	2	1	1	1	1	1	1
Duruturk 2019	2	1	1	1	1	1	1
Erdogan 2021	2	1	1	2	1	1	1
Gerceker 2016	2	1	1	2	1	1	1
Ghanbari 2021	2	1	1	3	1	1	2
Ghawadra 2020	2	1	1	3	1	1	2
Gu 2021	2	1	1	3	1	1	2
Guo L 2020	2	1	1	3	1	1	2
Guo y 2020	2	1	1	3	1	1	2
Hamedi 2020	2	1	1	2	1	3	3
Hatipoglu 2018	2	1	1	2	1	1	1
Heim 2021	3	1	1	1	1	3	3
Hua 2015	2	1	1	3	1	1	2
Huang 2018	2	1	1	2	1	1	1
Huang L 2021	2	1	1	1	1	1	1
Imamura 2021	2	1	1	2	1	1	1
Inangil 2020	1	1	1	3	1	1	2
Jannati 2020	3	1	1	3	1	1	3
Jareethum 2008	2	1	1	2	3	1	3
Khushnood 2021	2	1	1	1	1	1	1
Korkmaz 2020	2	1	1	2	1	1	1
Li 2021	1	1	1	3	1	1	2
Liu H 2022	2	1	1	3	1	2	3
Liu Z 2021	2	1	1	3	1	1	2
Luo Y 2021	3	1	1	2	1	1	2
Luo YJ 2021	1	1	1	2	1	1	1
Majd 2020	3	1	1	2	1	1	2
Mak 2015	3	1	3	2	1	3	3
Mehri 2020	2	1	1	2	1	1	1
Milani 2015	2	1	1	2	1	1	1
Moeini 2019	2	1	1	2	1	1	1
Mogoase 2013	2	1	1	2	1	1	1
Newman 2021	3	1	1	2	1	3	3
Ngai 2015	2	1	1	2	1	1	1
Nobakht 2020	2	1	1	2	1	1	1
Ofoegbu 2020	2	1	1	2	1	3	3
Osborn 2020	2	1	1	2	1	1	1

Pakrad 2021	2	1	1	2	1	1	1
Peng 2018	2	1	1	2	1	1	1
Rad 2018	2	1	3	2	1	1	3
Rahimi 2021	2	1	1	2	1	1	1
Salamanca-Sanabria 2020	1	1	1	2	1	3	2
Shahdosti 2020	3	1	1	2	1	1	2
Sivrikaya 2021	2	1	1	1	1	1	1
Song 2021	2	1	1	2	1	1	1
Srivastava 2020	2	1	1	2	1	1	1
Stamm 2018	2	1	1	2	1	2	2
Su 2021	2	1	1	2	1	1	1
Taleban 2016	2	1	3	2	1	1	3
Tam 2020	2	1	1	2	1	1	1
Thitipitchayanant 2018	1	1	1	2	1	1	1
Tiburcio 2018	2	1	1	3	1	3	3
Tol 2020	1	1	1	2	1	1	1
Torabizadeh 2021	2	1	1	2	1	1	1
Tulbure 2018	3	1	1	2	1	3	3
Tulbure 2015	3	1	1	3	1	1	3
Wang 2020	3	1	1	2	1	3	3
Wantanakorn 2018	2	1	1	2	1	1	1
Wei 2020	2	1	1	3	1	1	2
Xia 2020	2	1	1	2	1	1	1
Yan 2022	2	1	1	2	1	1	1
Yang B 2021	2	1	1	2	1	1	1
Yang L 2019	2	1	1	2	1	1	1
Yang M 2019	2	1	1	2	1	1	1
Yardimci 2019	2	1	1	2	1	1	1
Yeung 2018	2	1	1	3	1	1	2
Zengin 2021	2	1	1	1	1	1	1
Zhang QL 2021	2	1	1	2	1	1	1
Zhang QL 2021	2	1	1	3	1	1	2
Zhang X 2021	2	1	3	1	1	1	2
Zhang Y 2021	2	1	1	2	1	1	1
Zhao 2021	3	1	1	2	1	2	3
Zheng 2021	1	1	1	1	1	1	1
Zhianfar 2020	2	1	1	2	1	1	1
Zhou 2019	2	1	1	1	1	1	1
Zhuang 2017	2	1	1	2	1	3	3
