# Supplementary material for: Effectiveness of Digital Mental Health Tools to Reduce Depressive and Anxiety Symptoms in Low- and Middle-Income Countries: Systematic Review and Meta-analysis
Source: JMIR Ment Health. 2023 Mar 20;10:e43066. doi: 10.2196/43066 (PMC10131603; doi:10.2196/43066)
Supplement: Multimedia Appendix 3 [file mental_v10i1e43066_app3.pdf]

Multimedia Appendix 3. Grading of Recommendations Assessment, and Evaluation (GRADE) Assessment for the overall quality of evidence

Risk of bias
The risk of bias in the studies was not considered high in general regarding randomization, blinding, attrition, study design, and selective reporting.

- The random allocation sequence was properly described (n=72, 75%), and the described methods were appropriate (n=72, 100%). Not every study reported allocation concealment methods, but when they were reported, it was appropriate. Participants were blinded in 64% (n=61) of studies, and assessors were blinded in 54% (n=52) of studies.
- About 77% (n=74) of studies reported that the loss to follow-up rate is 0-20% and 65% (n=62) studies performed intention-to-treat analysis.
- No selective outcome reporting was detected because the outcomes of our interest were either primary or secondary outcomes, which were all reported.
- Non-standard RCTs, including cluster or crossover RCT designs, were none.

Inconsistency
Study outcomes were considered consistent overall in terms of the results of systematic review (conclusions were consistent) and meta-analysis (low variations of effect sizes). Although heterogeneity was considerable, the likely sources of heterogeneity were comprehensively suggested.

- Out of 96 studies included in the systematic review, 92 (96%) concluded that the intervention was effective on depression/anxiety outcomes, and 4 (4%) concluded that the intervention showed no effect on depression/anxiety outcomes.
- Effect estimation across the studies does not vary widely. The confidence interval of the overall effect estimation is narrow (Hedges' g= -0.61[-0.78 to -0.44] depression, Hedges' g = -0.73 [-0.93 to -0.53] anxiety).
- Approximately 69% of studies' effect sizes overlapped with the confidence interval of the overall effect estimate.
- Reported I^2 is considerable. However, the likely sources of heterogeneity were comprehensively explained by subgroup analysis and meta-regression results. Subgroup analysis showed intervention content, type, depression/anxiety level, outcome, and age could be the likely sources of heterogeneity for both depression and anxiety. Additionally, the meta-regression results signaled that the study region and the symptom level could be strong likely sources of heterogeneity for anxiety. Because the likely sources of heterogeneity were not reported in prior literature, this is the added value of the study.

Indirectness
Indirectness is low in this study as all the outcomes can directly answer our research question.
Q. Do digital mental health tools reduce depressive and anxiety symptoms in low- and middle-income countries?

- All the included studies can directly answer the study question in 4 categories (population, intervention, comparator, outcomes) because if the study did not meet the pre-specified criteria, those were excluded. 1) population: people in Low-and Middle-Income Countries, 2) intervention: digital mental health tools, 3) comparator (any comparison including no

intervention, usual care, active control), 4) outcomes (depression/anxiety symptoms either as primary or secondary outcomes).

Imprecision

Imprecision is considered low because the overall effect estimate was calculated from a large number of total participants (13,259) and was precise according to GRADE guidelines.

- The meta-analysis included 81 studies and contained a large number of study participants, a total of 13,259 participants (6,673 in technology intervention and 6,586 in the control group).
- For both depression and anxiety, the upper and lower limits of confidence intervals are on the same side (negative), meaning the intervention effectively decreases the depression and anxiety symptoms (consistently beneficial).
- For both depression and anxiety, neither the upper nor lower limits of the confidence intervals crossed the overall effect sizes of 0.5; Hedges' g= -0.61 (-0.78, -0.44) in depression, Hedges' g= -0.73 (-0.93, -0.53) in anxiety.

Publication bias

Publication bias is not considered significant to downgrade.

- 17 studies were considered small-size studies (total participants are less than 60) out of a total of 81 studies, as we included some pilot RCTs. However, all the small studies satisfied their sample size requirements for statistical significance.
- Funnel plots appeared to be symmetrical for both anxiety and depression. Egger's test results were not significant for depression (p-value=0.19) but significant for anxiety (p-value=0.03), suggesting potential publication bias for anxiety. However, when we ran Duval and Tweedie's trim-and-fill analysis to statistically assess the publication bias, no imputation was necessary to adjust for the publication bias in both depression and anxiety, and the effect sizes stayed the same.
