# Supplementary material for: Effectiveness of Digital Mental Health Tools to Reduce Depressive and Anxiety Symptoms in Low- and Middle-Income Countries: Systematic Review and Meta-analysis
Source: JMIR Ment Health. 2023 Mar 20;10:e43066. doi: 10.2196/43066 (PMC10131603; doi:10.2196/43066)
Supplement: Multimedia Appendix 5 [file mental_v10i1e43066_app5.pdf]

Multimedia Appendix 5. Characteristics of studies included in the systematic review.

First author	Region[a]	Population	Condition addressed (psycho-metric tool)[b]	Study groups	Intervention details for study groups
Abbasi 2021 [27]	Pakistan	Pediatric dental patients	Anxiety (Anx); (FIS)	(1) Intervention: mobile application 'Little Lovely Dentist' (n=40, age[m=6.8, sd=2.1]), (2) intervention: dental song (n=40, age[m=8.2, sd=2.3]), (3) control: no intervention (n=40, age[m=7.3, sd=1.7])	(1) The application "little lovely dentist": activities including restorations, fissure sealants, extractions, brushing, and playfully explaining oral hygiene methods to the child. (2) Dental song (3) No behavior modification technique
Adewu-ya 2019 [28]	Nigeria	Patients with depression	Depression (Dep): (PHQ-9)	(1) Intervention: mobile telephone supported Collaborative Stepped Care (mCSC) (n=439), (2) control: ordinary Collaborative Stepped Care (oCSC) (n=456)	(1) Mobile telephone– supported Collaborative Stepped Care: oCSC (psychological sessions and medication) with the addition of mobile telephone support to provide tailored information and reminders. (2) oCSC: manualized CSC intervention by trained staff (psychoeducation, problem-solving Therapy in Primary Care and medication).
Ahorsu 2020 [29]	Iran	Patients diagnosed with epilepsy with moderate insomnia	Anx and Dep (HADS)	(1) Intervention: iCBT (n=160, age[m=38.0, sd=9.9]), (2) control: standardized patient education (n=160, age [m=38.4, sd=13.5])	(1) iCBT (social and physical environmental reconstruction, sleep hygiene information, habit formation/reversal, action planning, problem solving, and self-monitoring). (2) Standardized patient education on sleep hygiene practices, healthy sleep behaviors,

					and changing lifestyle to promote healthy sleeping behaviors
Alessi 2021 [30]	Brazil	Adult patients with type 2 diabetes	Mental health disorder :dep/anx (SQR-20)	(1) Intervention: telehealth (n=46, age[m=61.6, sd=9.2]), (2) control: active control (n=45, age[m=61.0, sd=9.0])	(1) Telehealth: phone calls on individualized topics including mental health, physical activity, healthy eating, educational skills on healthy lifestyles, and complementing patients' usual clinical care (eg, glycemic control). (2) Active control: access a website (eg, diabetes care, mental health, and lifestyle habits)
Araya 2021 [31]	Brazil /Peru	Patients with depression	Dep (PHQ-9)	(1) Intervention: digital intervention (n=596), (2) control: enhanced usual care (n=604)	(1) Low-intensity digital intervention was delivered via a smartphone based on behavioral activation principles and supported by nurse assistants. (2) Enhanced usual care: depressive symptoms assessment and follow-ups up to 4 times.
Arjadi 2018 [26]	Indonesia	Patients with depression	Dep (PHQ-9)	(1) Intervention: online behavioral activation with lay support (Guided Act and Feel Indonesia, GAF-ID) (n=159, age[m=24.5, sd=4.93]), (2) control: online psychoeducation (n=154, age[m=24.5, sd=5.22])	(1) Guided Act and Feel Indonesia: weekly web-based module adapted from original Dutch behavioral activation intervention (Act and Feel). Monitoring daily mood and activities, encouraging users to do mood-independent activities (30-45 min) with the lay counselor supports. (2) Web-based basic psychoeducation without guidance or support.

Asadza-deh 2020 [32]	Iran	Women with traumatic childbirth experience	Dep (EPDS)	(1) Intervention: midwife-led counseling (n=44), (2) control: postnatal routine care (n=43)	(1) Face-to-face counseling session within 72 h after giving birth plus a telephone counseling 4-6 wks after giving birth (2) Postnatal routine care
Baruah 2021 [33]	India	Caregivers of Alzheimer disease or dementia	Dep (CES-D)	(1) Intervention: multi-component online caregiving support program (iSupport) (n=29, age [m=46.5, sd=14.1]), (2) control: education (n=26, age[m=24.5, sd=5.2])	(1) iSupport: a multicomponent web-based caregiver skills training and support program of the World Health Organization (2) Education-only ebook program
Byonan-ebye 2021 [34]	Uganda	People living with HIV	QOL_ Anx and Dep (MOS-HIV)	(1) Intervention: interactive voice response (IVR)-based community health (n=256), (2) control: standard of care (n=260). Overall age (median=32, IQR 25-40)	(1) Call for Life Uganda, an interactive voice response technology that is based on the Mobile Technology for Community Health open-source software: daily adherence and preappointment reminders, health information tips and option to report symptoms. (2) Standard of care comprising face-to-face facility appointments.
Chan KL 2019 [35]	Hong Kong	First-time mothers	Dep (EPDS)	(1) Intervention: smartphone-based antenatal education (n=330, age[m=31.3, sd=4.6]), (2) control: usual care (n=330, age[m=31.2, sd=4.5])	(1) A smartphone-based app (iParent) for antenatal education (2) Usual care: nurse-led antenatal program without access to an app (4 sessions).
Chavoo-shi 2017 [36]	Iran	People with medically unexplained pain	Anx and Dep (DASS-21)	(1) Intervention: internet-delivered Intensive Short-Term Dynamic Psychotherapy (ISTDP) (n=39, age[m=31.9,	(1) Internet-delivered ISTDP via Skype: psychotherapy emphasizing emotional awareness and augmenting the capacities to

				sd=6.2]), (2) control: in-person ISTDP (n=42, age[m=32.2, sd=6.2])	self-reflect as well as tolerate and experience emotions. (2) In-person ISTDP.
Chavoo-shi 2016 [37]	Iran	People with medically unexplained pain	Anx and Dep (DASS-21)	(1) Intervention: internet-delivered Intensive Short-Term Dynamic Psychotherapy (ISTDP) (n=50, age[m=38.8, sd=7.1]), (2) control: treat as usual (n=50, age[m=37.4, sd=6.8])	(1) Internet-delivered ISTDP via Skype: psychotherapy emphasizing emotional awareness and augmenting the capacities to self-reflect as well as tolerate and experience emotions. (2) Treat as usual.
Chiang 2017 [38]	Hong Kong	Main family caregivers of critically ill patients	Anx and Dep (DASS-21)	(1) Intervention: tablet-based caregiver education (Education of the Families by Tab, EF-T) (n=39), (2) control: usual care (Education of the Families by Routine, EF-R) (n=35)	(1) Education of the Families by Tab: more comprehensive and standardized nature of the information to be explained by intensive care unit nurses to the main family caregivers through audio-visual interactions with the tabs. (2) EF-R: routine information offered only through opportunistic verbal communication.
Ciuca 2018 [39]	Romania	People with panic disorder	Dep (PHQ-9)	(1) Intervention: guided iCBT (PAXonline program for panic disorder, PAXPD) (n=29, age[m=35.1, sd=11.7]), (2) intervention: unguided iCBT (n=25, age[m=35.3, sd=11.0]), (3) control: waitlist control (n=27, age[m=35.2, sd=8.4])	(1) Guided iCBT (PAXonline program for panic disorder). Real-time video session by a licensed psychotherapist for 15-45 min (2) Unguided iCBT (real-time video session) (3) Waitlist control
Constant 2014 [40]	South Africa	Women who did a medical	Anx and Dep (HADS)	(1) Intervention: text-message-based medication reminder and education	(1) A uniform program of automated text messages to remind of taking medication and

		abortion at home		(n=197, age[m=26.0, sd=5.6]), (2) control: standard of care (n=184, age[m=25.6, sd=5.4])	to inform on managing the bleeding, cramping, and side effects (such as pain, vomiting, and diarrhea) (2) Standard abortion care: abortion counseling, administration of mifepristone, misoprostol, and follow-up clinic visit.
Craveiro 2020 [41]	Brazil	Endodontics patients	Anx (VAS)	(1) Intervention: audio-visual based anxiety care (n=80), (2) control: no intervention (n=80)	(1) Audiovisual source (youtube.com) via a tablet computer (iPad) (2) No intervention
Cumino 2017 [42]	Brazil	Children undergoing elective surgery (4-8 y)	Anx (YPAS)	(1) Intervention: smartphone-based anxiety care (n=21), (2) control: usual information leaflet (n=21)	(1) Smartphone app: Galinha Pintadinha, I Go To Farm, Talking Tom, Cut the Rope, Where's My Water and Angry Birds (2) Information leaflet: 11 questions and answers (Q&As) commonly asked by the parents and 17 brief items related to anesthesia in children.
Digin 2022 [43]	Turkey	Patients who underwent cataract surgery	Anx (STAI)	(1) Intervention: SMS-based patient psychoeducation (n=41, age[m=57.2, sd=5.7]), (2) control: routine discharge care (n=41, age[m=58.0, sd=7.2])	(1) Texting-based patient psycho/education and after-surgery support including medication reminder and care instructions. (2) Routine discharge care: postoperative care education, including medicine intake information.
Duan 2018 [44]	China	Outpatients with coronary heart disease	Dep (CES-D)	(1) Intervention: internet-based behavioral modification (n=44), (2) control: waitlist control (n=39)	(1) Health Action Process Approach (HAPA theory–based internet behavior modification intervention, followed by behavior

					maintenance program regarding physical activity and healthy eating for 8 wk. (2) Waitlist control.
Duan 2017 [45]	China	Undergraduate students	Dep (CES-D)	(1) Intervention: web-based behavioral modification (n=88, age[m=19.0, sd=1.2]), (2) control: no intervention (n=54, age[m=19.4, sd=1.2])	(1) HAPA theory–based internet behavior modification intervention for physical activity and healthy eating targeting social cognitive indicators for 8 wks (2) No intervention
Duan 2022 [46]	China	College students	Dep (CES-D)	(1) Intervention: web-based physical exercise interventions (n=187, age[m=20.1, sd=1.1]), (2) control: placebo control (n=170, age[m=19.9, sd=1.1])	(1) Web-based physical exercise interventions: HAPA model-based risk perception, outcome expectancies, goal settings, development, and adjustment of action planning coping planning. (2) Placebo control: placebo treatment unrelated to physical activity and healthy eating
Duruturk 2019 [47]	Turkey	Patients with type 2 diabetes	Dep (BDI-II)	(1) Intervention: tele-rehabilitation (n=23, age[m=52.8, sd=11.9]), (2) control: usual care (n=21, age[m=53.0, sd=10.5])	(1) Tele-rehabilitation: 40 min at home by internet-based video conferences with the supervision of a physiotherapist. (2) Usual care: education plus their ongoing medical therapies.
Erdogan 2021 [48]	Turkey	Children undergoing venipuncture (7-12 y)	Anx (VAS)	(1) Intervention: distraction card (n=35, age[m=9.2, sd=1.6]), (2) intervention: virtual reality (n=37, age[m=9.5, sd=1.8]), (3) intervention: Buzzy (n=36, age[m=9.4, sd=1.7]), (4)	(1) Distraction card to distract children not to focus on anxious procedure. (2) VR: a 3D dinosaur animation, using a smartphone, VR glasses, and a headset for distraction. (3) Buzzy (local cold application and vibration to relieve pain) for distraction. (4)

				control: no intervention (n=34, age[m=9.4, sd=1.6])	No other nonpharmacological intervention.
Gercek-er 2016 [49]	Turkey	Parents of children (3-17 y), undergone outpatient surgery	Anx (STAI)	(1) Intervention: nurse-led telephone counseling (n=24, age[m=37.6, sd=6.2]), (2) control: no intervention (n=30, age[m= 38.6, sd=5.9])	(1) Nurse-led telephone counseling: each day after the outpatient surgery until they came to the clinic for a follow-up visit. Child follow-up regarding pain management, surgical site care, symptoms of infection, nutrition, sleep, and excretion. (2) No nurse-led telephone counseling.
Ghanbari 2021 [50]	Iran	Patients with nonmetastatic breast cancer	Anx (STAI)	(1) Intervention: mobile app-based psychoeducation (n=38), (2) control: waitlist control (n=39). Overall age(m=46.5, sd=9.3)	(1) Mobile app (BCSzone: guided self-management-based psychoeducation) and web-based social media–based support group intervention. (2) Waitlist control
Ghawa-dra 2020 [51]	Malaysia	Ward nurses	Anx (DASS-21)	(1) Intervention: social media-based mindfulness training (n=118), (2) control: waitlist control (n=106)	(1) Guided self-practice Mindfulness-Based Training web-based program (MINDFULGym). Social media (WhatsApp) was used for guidance, facilitation, and reminder. (2) Waitlist control
Gu 2021 [52]	China	Preoperated patients	Anx (APAIS and AVAT)	(1) Intervention: mobile app-based anxiety relief (n=40), (2) control: no intervention (n=40)	(1) A smartphone to watch a video from TikTok to distract from operation-related anxiety. (2) No intervention
Guo L 2020 [53]	China	Depressive symptomatic pregnant women	Anx (STAI), Dep (EPDS, BDI-II)	(1) Intervention: web-based psychological program (n=144, age[m=31.4, sd=5.7]), (2) control: no intervention (n=140, age[m=29.8, sd=6.2])	(1) A web-based psychological intervention adapted from Mindfulness and Compassion with Self and Others but focused on self-compassion: comprises training (10 h of training with 36 episodes), exercise, and self-

					reflection practice with diary. (2) No intervention
Guo y 2020 [54]	China	People living with HIV	Dep (CES-D)	(1) Intervention: mobile app-based mental health program (Run4Love) (n=150, age[m=28.0, sd=5.8]), (2) control: usual care (n=150, age[m=28.6, sd=5.9])	(1) Run4Love: stress reduction and coping skills (muscle relaxation, breathing, and meditation via multimedia format), physical activity (goal setting, personalized feedback. (2) Usual care [waitlist control]: a brochure on nutrition in addition to usual care for HIV treatment, information on healthy diet).
Hamedi 2020 [55]	Iran	Children with cancer	Anx (STAI), Dep (CDI)	(1) Intervention: cCBT (n=15), (2) intervention: no computerized CBT (n=15), (3) control: no intervention (n=15). Age range 9-12 years.	(1) Computerized CBT: psychoeducation and training were provided via texts, animations, and games. (2) No computerized CBT: 6 psychological in-person sessions. (3) No intervention.
Hatipog-lu 2018 [56]	Turkey	Children undergoing outpatient surgery	Anx (YPAS)	(1) Intervention: audiovisual presentation (n=33, age[m=7.6, sd=2.0]), (2) control: usual care (n=33, age[m=7.6, sd=2.3])	(1) An audiovisual presentation to inform about pre and postoperative care (eg, anesthesia management, preoperative fasting, medication after surgery). (2) Usual care (verbal information session)
Heim 2021 [57]	Lebanon	Depressive population affected by adversity	Anx (GAD-7), Dep (PHQ-9)	(1) Intervention: mobile app-based intervention (Step-By-Step) (n=24, age[m=28.2, sd=7.9]), (2) control: enhanced usual care (n=18, age[m=26.4, sd=8.0])	(1) Step-by-step (a short introduction with 5 sessions) plus ehelper support (nonspecialists who offer minimal guidance, 15 min weekly). (2) Enhanced usual care: one information session with

					the app plus text messages (basic psychoeducation).
Hua 2015 [58]	China	Children with chronic wounds on lower limbs (4-16 y)	Anx (VAS)	(1) Intervention: virtual reality (VR) (n=33, age[m=8.9, sd=3.4]), (2) control: usual care (n=32, age[m=8.6, sd=3.4])	(1) VR-based distraction: Ice Age 2, the meltdown game to achieve the virtual distraction activity (a 3D virtual environment). (2) Standard distraction methods such as toys, television, books, and parental comforting.
Huang 2018 [59]	China	Patient with chronic hepatitis B with late pregnancy	Anx (SAS), Dep (SDS)	(1) Intervention: routine care plus WeChat-based nursing care (n=45, age[m=33.5, sd=3.7]), (2) control: routine nursing care (n=45, age[32.8, sd=5.4])	(1) Routine care plus WeChat platform care for nursing (animated pictures, text, video clips, and voice), education (transmission route, development, and preventive treatment), communication (encouraging messages to boost mood, group feedback, and interaction) (2) Routine care: health education on mental health, medication, diet, and postdischarge guidance
Huang L 2021 [60]	China	First-time mothers during the early stage of motherhood	Dep (EPDS)	(1) Intervention: internet-based parenting support (n=20, age[m=27.2, sd=3.2]), (2) control: routine postnatal care (n=20, age[m=27.4, sd=3.0])	(1) Routine postnatal care plus internet-based Support Program (based on the self-efficacy theory and the social exchange theory, which has 5 components including learning forum, communication forum, ask-the-expert forum, baby home forum, and reminder forum). (2) Routine postnatal care: supports from the obstetricians, obstetric nurses, and community doctors (home visits) during postpartum.

Imamura 2021 [61]	Vietnam	Hospital nurses	Anx and Dep (DASS-21)	(1) Intervention: free choice app-based stress management (n=291, age[m=33.7, sd=7.3]), (2) intervention: fixed choice app-based stress management (n=293, age[m=32.8, sd=6.6]), (3) control: usual care (n=296, age[m=32.8, sd=6.4])	Two smartphone-based 6-module stress management programs. (1) Free-choice program: respondents selected one module per week in any order they preferred. (2) Fixed-choice program: respondents were required to study modules in a fixed order, one module per week. (3) Usual care: standard hospital assistance service (Waitlist control).
Inangil 2020 [62]	Turkey	School-aged children who have undergone phlebotomy	Anx (CFS)	(1) Intervention: virtual reality (n=40, age[m=9.3, sd=1.8]), (2) intervention: tablet-based cartoon watching (n=40, age[m=9, sd=1.7]), (3) control: no intervention (n=40, age[m=9, sd=1.7])	(1) VR group (VR box, a head-mounted display with stereo earphones. The cartoon image was transmitted onto the screen in front of the child's eyes). (2) Tablet group (children watched the cartoons on a 7.0-inch tablet computer). (3) No intervention.
Jannati 2020 [63]	Iran	Postpartum women	Dep (EPDS)	(1) Intervention: mobile app-based CBT (Happy Mom) (n=38, age[m=27.7, sd=4.2]), (2) control: waitlist control (n=37, age[m=27.4, sd=4.8])	(1) Happy Mom: CBT-based goal setting, emotional recognition, noticing thoughts, thought challenging, problem solving, improving social skills, relapse prevention. (2) Waitlist control
Jareeth-um 2008 [64]	Thailand	Pregnant women	N/A	(1) Intervention: text-message-based prenatal support (n=32), (2) control: usual care (n=29).	(1) Two SMS text messages per week: information about common symptoms during pregnancy and health education appropriate to the women's gestational age. (2) No SMS text message, but both groups had the same antenatal and perinatal care.

Khushn-ood 2021 [65]	Pakistan	Older adult population	QOL_ Dep (EQ-5D-5L)	(1) Intervention: exer-game-based balance training (n=42), (2) control: conventional balance training (n=41)	(1) Indulged in gaming via Wii fit (Nintendo Co, Ltd) for 30 min twice a week which included basic step, basic run, soccer heading, table tilt, balance bubble, and lotus focus. (2) Conventional balance training (eg, single-leg stand up, tandem walk, and contralateral arm raise)
Korkm-az 2020 [66]	Turkey	People undergoing Mammaplasty	Anx (STAI)	(1) Intervention: web-based patient education (n=22), (2) intervention: brochure-based education (n=22), (3) control: standard of care (n=22)	(1) Web-based patient education (preoperative preparation, exercises, postoperative care, therapies, and breast self-examination). (2) Brochure-assisted education (the same content of the website). (3) Standard verbal education.
Li 2021 [67]	China	Men who had sex with men	Anx (GAD-7), Dep (CES-D)	(1) Intervention: social media-based intervention, three good things (TGT-SN) (n=129), (2) intervention: TGN only (n=139), (3) control: active control (health education) (n=136)	(1) TGT-SN: to post brief messages to the group every day three good things that they had experienced or felt grateful, to read others' posts and provide at least three feedbacks on those. (2) TGN only: to write messages daily three good things that they had experienced or felt grateful. (3) Mental health education from the research assistants via QQ messaging app.
Liu H 2022 [68]	China	University students	Anx (GAD-7), Dep (PHQ-9)	(1) Intervention: chatbot-delivered CBT (n=33, age[m=23.4, sd=1.8]), (2) control: active control	(1) Chatbot-delivered intervention (XiaoNan) via WeChat: chatbot provided CBT-based strategies and responded empathetically to

				(bibliotherapy) (n=30, age[m=22.8, sd=1.7])	the user's random conversation. (2) Bibliotherapy: read literature and follow psychological interventions from the literature to alleviate their problems.
Liu Z 2021 [69]	China	Patients with COVID-19	Anx (HAMA, SAS), Dep (HAMD17)	(1) Intervention: cCBT (n=126, age[m=43.8, sd=14.3]), (2) control: treat as usual (TAU) (n=126, age[m=41.5, sd=11.5])	(1) TAU+ cCBT: systematically intervene in patients' cognition, emotions, and behavior through computer-based, visually attractive, and interactive examples, exercises, and videos. (2) TAU: psychological assessments and support and consultations for overall well-being.
Luo Y 2021 [70]	China	Parents of children with cancer	Dep (SDS)	(1) Intervention: web-based parental resilience training program (n=52), (2) control: standard education (n=51). Overall age (m=33.6, sd=5.2)	(1) Web-based parental resilience training program: to read and watch the tweet sessions, finish the web-based assignments, and receive feedback from a psychological consultant via WeChat. (2) Standard education: child cancer care manual (oral care, symptom management, central catheter maintenance, dietary and medication guidance, and infection prevention).
Luo YJ 2021 [71]	China	Body-dissatisfied young Chinese women	Dep (CES-D)	(1) Intervention: internet dissonance-based eating disorder prevention (eBody Project) (n=121), (2) control: active control (brochure education) (n=128). Overall age(m= 17.4, sd= 1.4)	(1) eBody Project: to critique the feminine attractiveness idea and increase self-acceptance via a user-driven self-education exercise, an educational game, and an offline exercise. (2) Education brochure: descriptions

					of positive and negative body image (eg, increased risk for eating disorder onset).
Majd 2020 [72]	Iran	Patients with insomnia	Anx and Dep (HADS)	(1) Intervention: mobile app-based theory-based cognitive behavioral technique (n=156, age[m=36.2, sd=5.8]), (2) control: active control (n=156, age[m=35.3, sd=5.8])	(1) Several theories (CBT, theory of planned behavior , HAPA, and control theory are integrated for information about health consequences, habit formation and reversal, reconstructing the social and physical environment, self-monitoring of behavior, action planning, and problem solving. (2) Received written information on insomnia symptoms, physiological controls of sleep, sleep hygiene practices, healthy sleep behaviors, and changing lifestyles.
Mak 2015 [73]	Hong Kong	General public (university students and staff)	Dep (DASS-21)	(1) Intervention: Health Action Process Approach (HAPA)-based web mindfulness (n= 105), (2) intervention: basic web mindfulness (n=104), (3) control: waitlist control (n=79). Overall age(m=22.8, sd=6.5)	(1) HAPA-based web mindfulness: basic mindfulness course materials plus additional guidance derived from the HAPA model to help them translate intention into action and keep up the exercises (eg, action, maintenance, and recovery of self-efficacy, action, and coping planning). (2) Basic mindfulness material. (3) Waitlist control.
Mehri 2020 [74]	Iran	Parents of children with attention	Anx and Dep (DASS-21)	(1) Intervention: text-message-based parenting	(1) Group-based behavioral parent therapy+ telephone follow-ups: information about sleep

		deficit/ hyperactivity disorder		training (n=28), (2) control: waitlist control (n=27)	disturbance, behavioral principles, nutritional health, developing beneficial boundaries for children, controlling children's environmental triggers, and problem-solving methods (2) Waitlist control
Milani 2015 [75]	Iran	Postpartum women	Dep (EPDS)	(1) Intervention: telephone-based support (n=22, age[m=27.6, sd=4.8]), (2) control: usual care (n=24, age[m=28.4, sd=7.4])	(1) Telephone support by health volunteers: 2-3 times calls weekly for 6 wks to check mother's health and relationship with the baby and husband and newborn's condition+routine postpartum care. (2) Routine postpartum care: education and counseling, nutrition advice without mental health care.
Moeini 2019 [76]	Iran	Female adolescents	Dep (CES-D)	(1) Intervention: web-based intervention (Dorehye Amozeshie Dokhtaran, DAD) (n=48, age[m=16.2, sd=0.7]), (2) control: no intervention (n=60, age[m=16.5, sd=0.6])	(1) Dorehye Amozeshie Dokhtaran: to identify their own unproductive beliefs and convert these into reasonable and beneficial thinking. Participants received SMS text message reminders, shared experiences, asked questions, and responded to others in the group. (2) No intervention
Mogoase 2013 [77]	Romania	Depressed undergraduate students	Dep (BDI-II)	(1) Intervention: internet-based concreteness training (n=20), (2) control: waitlist control (n=21). Overall age (m=22.9, sd=4.3)	(1) Participants received daily (via email) 2 standard forms describing hypothetical events—1 positive and 1 negative. They were instructed to read every scenario and spend at least 2 min

					concentrating on it while trying to visualize the "movie" of that possible event as if they were in it. (2) Waitlist control
Newman 2021 [78]	India	University students with generalized anxiety disorder	Anx (GAD-Q-IV), Dep (DASS-21)	(1) Intervention: technology-based guided self-help CBT (n=117, age[m=20.0, sd=1.7]), (2) control: waitlist control (n=105, age[m=19.7, sd=1.4])	(1) Guided Self-Help Intervention via any internet-enabled computer, mobile phone, or tablet, based on CBT: behavior change, automatic thoughts, cognitive reframing, situational exposure, mindfulness, and habit formation. Supported by an assigned coach via text messages and telephone consultation (2) Waitlist control
Ngai 2015 [79]	Hong Kong	Postpartum women	Dep (EPDS)	(1) Intervention: telephone-based CBT (n=197), (2) control: standard of care (n=200)	(1) Telephone-based CBT: to identify and modify depressive thoughts, enhance effective problem solving strategies and decision-making skills to deal with practical issues of childcare and common neonatal problems, and manage interpersonal difficulties by an experienced midwife. (2) Standard of care: 6-wk postpartum follow-up. Women can seek services and advice from maternal and child health centers.
Nobakht 2020 [80]	Iran	Mothers of children with cerebral palsy	Anx and Dep (DASS-21)	(1) Intervention: web-based daily care training (n=42, age[m=34.2, sd=5.9]), (2) control: routine occupational therapy (n=49, age[m=33.2, sd=5.7])	(1) Web-based intervention for daily care training: interaction with the system, an occupational therapist, and peers on the web (feeding, bathing, movement principle, sleep, play, and

					caregiver self-care) (2) Routine occupational therapy
Ofoegbu 2020 [81]	Nigeria	University students with depression	Dep (BDI-II)	(1) Intervention: guided internet-assisted intervention (GIAI) (n=56, age[m=24.2, sd=5.2]), (2) control: usual care (n=58, age[m=23.8, sd=4.4])	(1) Guided internet-assisted intervention is a structured and standardized intervention delivered through the internet: psychoeducation via internet and therapist-support via telephone and internet. (2) Usual care.
Osborn 2020 [82]	Kenya	High-school students	Anx (GAD-7), Dep (PHQ-8)	(1) Intervention: internet-based intervention (Shamiri-Digital) (n=50, age[m=15.4, sd=1.2]), (2) control: active control (n=53, age[m=15.7, sd=1.2])	(1) Shamiri-Digital: growth mindset (eg, academic, interpersonal, and personality traits), gratitude (eg, Three Good Things), value affirmation (eg, affirming personal values, virtues). (2) Study-skills control: note-taking skills and effective study habits which required similar effort to the intervention.
Pakrad 2021 [83]	Iran	Patients who have undergone coronary artery bypass surgery	Anx and Dep (DASS-21)	(1) Intervention: mobile app-based hybrid cardiac rehabilitation (CR) (n=39, age[m=62.6, sd=8.1]), (2) control: usual care(n=42, age[m=62.9, sd=9.8])	(1) Traditional CR (8 in-person, 4 group sessions, 2 mo) followed by remote care (24 discussion sessions in cardiac disease, control of medical and lifestyle risk factors, medications via smartphone app, 3 mo). (2) Usual care: 1-mo traditional CR (8 in-person, 4 group sessions).
Peng 2018 [84]	China	Patients with heart failure	Anx and Dep (HADS)	(1) Intervention: telehealth exercise (n=42), (2) control: usual care (n=41). Overall age (m=66.3, sd=10.5)	(1) Home-based exercise training via telephone, instant messaging follow-ups, and consultations by a multidisciplinary team (physiotherapists for exercise,

					cardiac nurses for follow-up and self-care, and psychiatric nurses for psychological care). (2) Usual care: simple discharge education and regular follow-up.
Rad 2018 [85]	Iran	Social media–addicted people	Anx (BAI) Dep (BDI)	(1) Intervention: specially designed mobile app for social networking addicted people (n=100), (2) control: no intervention (n=100).	(1) Mobile app for social networking for Q&A session in the beginning of the treatment and at the end of second week of treatment. (2) No intervention.
Rahimi 2021 [86]	Iran	Women with failed in vitro fertilization cycles	Anx and Dep (DASS-21)	(1) Intervention: hope-oriented counseling with telephone follow-up (n=26, age[m=34.4, sd=6.0]), (2) control: usual care (n=26, age[m=32.9, sd=7.4])	(2) Hope-oriented counseling: in groups of 7-8 in six 45-60-min sessions weekly and telephone follow-up to remind the next session and ask questions (eg, express feelings, share concerns, care mental health). (2) Usual care (Waitlist control).
Salaman-ca-Sanabria 2020 [87]	Colombia	Colombian college students	Anx (GAD-7), Dep (PHQ-9)	(1) Intervention: culturally adapted iCBT (n=21, age[m=22.2, sd=5.4]), (2) control: waitlist control (n=54, age[m=22.1, sd=3.9])	(1) Culturally adapted iCBT: "Yo puedo sentirme bien"(I can feel better) is a modified version of the clinically efficacious Space from Depression: self-monitoring, behavioral activation, and challenging core beliefs via videos, and activities with the assigned supporter. (2) Waitlist control.
Shahdo-sti 2020 [88]	Iran	Patients in intensive care unit	Anx and Dep (HADS)	(1) Intervention: online family visitation (n=33, age[m=51.8, sd=13.5]), (2) control: usual care (n=33, age[m=50.8, sd=15.1])	(1) Web-based video visitations after surgery: two times a day on the second and the third day after surgery. All the patients had at least two web-based video

					visitations per day with their families. (2) Routine ward care without family visitation.
Sivrika-ya 2021 [89]	Turkey	Patients undergoing lower third molar extraction	Anx (STAI)	(1) Intervention: social media-based communication with the providers (n=36, age[m=27.0, sd=3.8]), (2) control: no intervention (n=36, age[m=26.9, sd=3.8])	(1) -1. Social media–based communication with the providers after the operation. (1)-2. Social media–based communication before the operation. (1)-3. Social media–based communication both before and after the operation. (2) No communication opportunity.
Song 2021 [90]	Iran	Patients with ankylosing spondylitis	Dep (BDI-II)	(1) Intervention: social media-based education (n=59, age[m=30.8, sd=8.8]), (2) control: standard of care (health advice) (n=59, age[m=29.1, sd=7.6])	(1) WeChat-based educational intervention: 20-30 min of video/voice calls via WeChat about basic knowledge, exercise, medication, daily life management, psychological support, and self-assessment. Additional web-based support via active Q&As (12 wks). (2) Standard of care: basic health advice and brief guidance on medication and exercise.
Srivasta-va 2020 [91]	India	Adolescents with mental health issues	Dep (BDI-II)	(1) Intervention: cCBT (Smartteen) (n=10, age[m=16.0, sd=1.5]), (2) control: treat as usual (n=9, age[m=16.1, sd=1.3])	(1) Smartteen: cCBT (12 wk). (2) Treatment as usual: pharmacotherapy and psychological therapy, including psychoeducation, supportive program, and stress management.
Stamm 2018 [92]	Brazil	Radiotherapy outpatients	Anx (STAI)	(1) Intervention: telephone-based intervention (anxiety care and education) (n=20, age[m=62.2, sd=11.8]), (2)	(1) Telephone call for 15 min: specific information on radiotherapy and Q&A, sharing concerns about side effects. (2)

				telephone-based active control (follow-up only) (n=19, age[m=63.0, sd=10.3])	Telephone call for 3 min: appointment confirmation and information about fluid intake.
Su 2021 [93]	China	Hospitalized patients with coronary heart disease	Anx and Dep (DASS-21)	(1) Intervention: nurse-led eHealth Cardiac Rehabilitation (NeCR) (n=73, age[m=55.5, sd=7.3]), (2) control: usual care (n=73, age[m=56.0, sd=7.0])	(1) Nurse-led eHealth Cardiac Rehabilitation: to optimize cognitive (ie, knowledge), behavioral (eg, self-efficacy) and environmental factors (eg, peer support) guided by social cognitive theory, and to empower patients' goal-attainment process via web sites and social media. (2) Usual care: education on medication use and lifestyle changes (physical activity, diet and smoking cessation).
Taleban 2016 [94]	Iran	Depressive individuals in rural area	Dep (BDI-II)	(1) Intervention: bibliotherapy with text-message (n=67, age[m=39.7, sd=9.2]), (2) intervention: bibliotherapy (n=66, age[m=39.6, sd=9.5]), (3) control: no intervention (n=65, age[m=41.4, sd=10.4])	(1) Bibliotherapy and text message: booklet (depression signs and symptoms, psychotherapy, negative thoughts, coping strategies against insomnia) and daily text messages (words of wisdom). (2) Bibliotherapy: booklet. (3) No intervention.
Tam 2020 [95]	China	Migrant children in Beijing	Dep (CES-D)	(1) Intervention: resilience-based psychoeducation via interactive games, videos, rehearsals, role-playing (n=121), (2) control: waitlist control (n=155)	(1) To promote psychosocial well-being and enhance protective factors against acculturative stressors; Activating-event-Belief-Consequence (ABC) model-based positive reinforcement, and psychological education (80 min) via interactive games, videos,

					role-playing, and drawing. (2) Waitlist control.
Thitipit-chaya-nant 2018 [96]	Thailand	Nulliparous blues mothers	Stein's postpartum blues score	(1) Intervention: self-help relaxation (Self-EAR) (n=39, age[m=23.7, sd=3.8]) , (2) control: routine postnatal care (n=37, age[m=23.8, sd=4.3])	(1) Self-empowerment-affirmation-relaxation: self-empowerment and self-affirmation-based relaxation program. Relaxation audio files were downloaded to the participants' digital device for home practice 3 times a day. (2) Routine postnatal care.
Tiburcio 2018 [97]	Mexico	Individuals seeking outpatient treatment for substance use	Dep (PHQ-9)	(1) Intervention: web-based help program for drug abuse and depression (PAADD www.paadd.mx) (n=12), (2) control: treat as usual (n=10)	(1) PAADD: based on the transtheoretical model of change and used CBT approach (self-control techniques, risk factor identification, and coping skills). Additional support for motivation by a counselor through a messaging system (8 wk). (2) Treatment as usual: weekly ordinary treatment (face-to-face CBT).
Tol 2020 [98]	Uganda	South Sudanese refugees in Rhino Camp settlement in Uganda	Dep (PHQ-9)	(1) Intervention: CBT-based self-help program via audio files (n= 283, age[m=30.9, sd=10.3]), (2) control: enhanced usual care (n=330, age[m=31.0, sd=11.4])	(1) Self-Help Plus: based on ACT, CBT elements (eg, engagement and psychoeducation), and specific techniques (cognitive defusion, mindfulness, and value clarification). Audio-recording is delivered by nonspecialists, facilitating scripted individual exercises and small group discussions. Plus, enhanced usual care. (2) Enhanced usual care:

					psychoeducation and psychological support.
Torabiz-adeh 2021 [99]	Iran	Patients undergoing coronary artery angiography	Anx and Dep (DASS-21)	(1) Intervention: multi-media and text-message-based patient education (n=40, age[m=54.9, sd=9.6]), (2) intervention: multimedia-based education (n=40, age[m=56, sd=9.1]), (3) control: usual care (n=40, age[m=54.6, sd=10.4])	(1) Text messaging and multimedia/DVD-based patient education. (2) Multimedia-based education (related pictures, animations, 15 min). (3) Usual care: traditional printed pamphlet. Contents were the same for all groups: what to do before angiography, how angiography is performed, and medication intake.
Tulbure 2018 [100]	Romania	Clinically depressed participants	Dep (BDI-II)	(1) Intervention: religious framework-based iCBT (n=19, age[m=32.2]), (2) intervention: conventional iCBT (n=34, age[m=29.2]), (3) control: waitlist control (n=26, age[m=35.7])	(1) Religious framework-based iCBT (behavioral activation, cognitive restructuring, forgiveness, altruism, and gratitude were facilitated via Christianity value). (2) Conventional iCBT. (3) Waitlist control.
Tulbure 2015 [101]	Romania	Patients with social anxiety disorder	Dep (BDI-II)	(1) Intervention: iCBT (iSOFIE) (n=38, age[m=30.6, sd=8.0]), (2) control: waitlist control (n=38, age[m=27.9, sd=7.8])	(1) iCBT (iSOFIE): participants were asked to fill in essay questions, provide thought records, build anxiety hierarchies, describe their exposure exercises, and complete a weekly social anxiety measure. Web-based support by psychologist was available. (2) Waitlist control.
Wang 2020 [102]	China	Patients with social anxiety disorder	Anx (SIAS)	(1) Intervention: self-help iCBT (n=47, age[m=25.9, sd=4.3]), (2) intervention: guided iCBT (n= 33,	(1) Self-help iCBT (culturally adapted motivation arousing, relaxation training, psychoeducation, cognitive

				age[m=24.7, sd=5.4]), (3) control: waitlist control (n=24, age[m=23.3, sd=3.6])	construct, attention training, and problem solving). (2) Guided iCBT. (3) Waitlist control.
Wantan-akorn 2018 [103]	Thailand	Children undergoing bone marrow aspiration (5-12 y).	Anx (mYPAS)	(1) Intervention: mobile app-based patient education and audio-visual activity (n=30, age[m=9.2, sd=3.4]), (2) control: usual care (n=30, age[m=9.6, sd=3.1])	(1) Patient education with a short, animated video about the bone marrow aspiration procedure according to the preoperational and concrete operational stage of school-aged children's cognitive development level. (2) Usual care.
Wei 2020 [104]	China	Patients with laboratory-confirmed COVID-19 in the isolation ward	Anx (HAMA), Dep (HAMD17)	(1) Intervention: internet-based self-help program (n=13, age[m=40.8, sd=13.5]), (2) control: daily supportive care (n=13, age[m=48.5, sd=9.5])	(1) The internet-based self-help intervention containing four main components: breath relaxation training, mindfulness (body scan), "refuge" skills, and butterfly hug method. (2) Daily supportive care only.
Xia 2020 [105]	China	People who have underwent colostomy	Anx (STAI)	(1) Intervention: social media and telephone-based continuous care (n=81), (2) control: standard of care (n=74)	(1) Self-management manual for colostomy care from hospital plus effective communications for the caregivers with the colostomy therapist via WeChat, blog, telephone, QQ to check and report the patients' symptoms and mood. (2) Standard of care: information on self-care of the colostomy stoma and bags and lifestyle-based advice.
Yan 2022 [106]	China	Parents of premature infants	Anx (SAS), Dep (SDS)	(1) Intervention: social media-based remote follow-up management (n=75, age[m=29.6, sd=7.5]), (2) control: routine outpatient	(1) WeChat-based remote follow-up management for education (premature infants care and feeding) and communication (Q&A with a medical staff 6:00

				follow-up management (n=75, age[m=28.1, sd=8.2])	PM-9:00 PM daily to care parents' concerns), and peer support through social media (share experience and discuss common topics). (2) Routine outpatient follow-up management.
Yang B 2021 [107]	China	Parents of premature newborns with patent ductus arteriosus	Anx (SAS), Dep (SDS)	(1) Intervention: multimedia-based communication and education (n=47), (2) control: treat as usual (n=47)	(1) WeChat-based post discharge follow-up: education (nursing and feeding, parents' psychological counseling) and communication (Q&A session with medical staff 6:00 PM-10:00 PM daily), and peer support. (2) Treat as usual: education pamphlet upon discharge.
Yang L 2019 [108]	China	Patients with coronary artery disease and depression	Dep (HADS, SDS)	(1) Intervention: patients' intensive telephone-based care program (PITC) (n=112, age[m=61.3, sd=8.6]), (2) control: usual care (n=112, age[m=60.9, sd=10.8])	(1) Patients' intensive telephone-based care program: weekly phone calls (30 min) in the first 6 mo and then biweekly for the subsequent 6 mo (communication to care concerns and provide disease care information). (2) Usual care: standard in-hospital treatment and regular examinations, general advice
Yang M 2019 [109]	China	Pregnant women	Anx (GAD-7), Dep (PHQ-9)	(1) Intervention: online mindfulness (n=52, age[m=31.3, sd=5.0]), (2) control: treat as usual (n=50, age[m=30.4, sd=3.9])	(1) Web-based mindfulness: theoretical constructions of mindfulness, attention monitoring, and acceptance theory for physical and emotional experiences, stress coping skills via text messages and audio files. (2) Treat as usual: antepartum health education for

					childbirth, breastfeeding, and parenting.
Yardim-ci 2019 [110]	Turkey	Patients with implantable cardioverter defibrillator	Anx (FSAS)	(1) Intervention: web-based intervention (n=139, age[m=46.2, sd=12.0]), (2) control: treat as usual (waitlist control) (n=137, age[m=50.9, sd=14.4])	(1) A web-based intervention (Living with an implantable cardioverter defibrillator): participants had discussions, shared their feelings and opinions with others, received educational information via pictures, figures, charts. (2) Treat as usual (Waitlist control).
Yeung 2018 [111]	China	Patients with depression	Dep (CES-D)	(1) Intervention: cCBT (n=29), (2) control: treat as usual (n=32). Overall age (m=33.0, sd=9.2)	(1) Usual care+MoodGYM (iCBT): 5 interactive modules (weekly), reminder emails, or phone calls to improve adherence. (2) Usual care (Waitlist control).
Zengin 2021 [112]	Turkey	Parents of young children during COVID-19	Anx (STAI)	(1) Intervention: online solution-focused support program (n=37), (2) control: no intervention (n=40). Overall age (m=33.5, sd=6.5)	(1) Web-based solution-focused support program through group discussion and homework assignments (journaling and breathing exercise) in getting acquainted, awareness of exceptions, and alternatives/coping skills/positive design of the future. (2) No intervention.
Zhang QL 2021 [113]	China	Parents of infants with congenital heart disease surgery	Anx (SAS), Dep (SDS)	(1) Intervention: telemedicine (n=42, age[m=29.1, sd=6.5]), (2) control: treat as usual (n=42, age[m=28.8, sd=7.1])	(1) Telemedicine via WeChat for health education (postoperative care, family care, and complication management), communication (Q&A session with medical staff 6:00 PM-9:00

					PM daily to care parent's concerns), and peer support via group interactions. (2) Treat as usual: information leaflet package
Zhang QL 2021 [114]	China	Parents of children with ventricular septal defects	Anx (SAS)	(1) Intervention: WeChat-based preoperative health education (n=35), (2) control: treat as usual (n=30)	(1) WeChat-based preoperative health education (preoperative care, family care, feeding, and complication management), communication (Q&A session with a medical staff 6:00 PM-10:00 PM daily to care parents' concerns), peer support via group interactions. (2) Treat as usual: leaflet package with the same information.
Zhang X 2021 [115]	China	Pregnant women	Anx (GAD-7), Dep (EPDS)	(1) Intervention: self-help mindfulness via social media (n=54, age[m=28.5, sd=3.7]), (2) control: health education (n=54, age[m=29.2, sd=3.5])	(1) Self-help mindfulness via social media: mindfulness courses (mindfulness-based stress reduction, cognitive therapy, and acceptance and commitment therapy), practices (a body scan and mindful breathing), daily audio file, and reminders via WeChat. (2) Health education for antenatal care via WeChat.
Zhang Y 2021 [116]	China	Sedentary young women	Anx (STAI)	(1) Intervention: online bodyweight high-intensity interval training (HIIT) (n=33, age[m=22.6, sd=2.2]), (2) control: waitlist control (n=29, age[m=22.8, sd=2.3])	(1) Home-based bodyweight high-intensity interval training – centered exercises (core, cardiopulmonary, muscular endurance training) and health education (body self-cognition, and diet/fat-reducing, shaping/stretching, meditation/relaxation, and

					emotion regulation) per week (6 wk) via video conference calls. (2) Waitlist control
Zhao 2021 [117]	China	Parents of children with autism spectrum disorder	Anx and Dep (DASS-21)	(1) Intervention: web-based parent–child physical activity via video conference calls (n=37, age[m=33.8, sd=3.9]), (2) control: waitlist control (n=38, age[m=33.4, sd=4.2])	(1) A web-based parent–child physical activity via video conference: warm-up, parent–child exercises, play-based exercises, cool-down and reward activities under the guidance and facilitation of trainers who were physical education teachers. (2) Waitlist control
Zheng 2021 [118]	China	School-aged children	Anx (SCAS)	(1) Intervention: online health information plus a peer-to-peer live-streaming (Recess and Exercise Advocacy Program, REAP) (n=485), (2) control: online health information (n=469)	(1) REAP plus web-based health education. REAP is a live streaming platform to capture short videos and photographs with smartphones. Participants were prompted by text messaging to participate in live streaming or posting their workouts videos. (2) Web-based health education: work out videos (no live streaming) and exercise programs at home, eye relaxation and stretch for 10 min
Zhianfar 2020 [119]	Iran	Hemodialysis patients	Dep (BDI-II)	(1) Intervention: CBT and telephone-based social support (n=33), (2) control: no intervention (n=33)	(1) CBT and telephone-based social support: relevant video tracks to encourage to comply with the therapeutic regimen, conduction of 8 CBT group sessions along with telephone-

					based peer support conciliation. (2) No intervention
Zhou 2019 [120]	China	Postsurgical women with breast cancer	Anx (SAS), Dep (SDS)	(1) Intervention: Cyclic Adjustment Training (CAT) and routine nursing care (n=66, age[m=44.6, sd=7.9]), (2) control: routine nursing care (n=66, age[m=44.4, sd=7.3])	(1) Cyclic Adjustment Training: training to enhance self-resilience via coping mechanism and adaptation for 12 wks. Plus, routine nursing care. (2) Routine nursing care: health education, vital signs, and postsurgery complications monitoring.
Zhuang 2017 [121]	China	Patients with functional dyspepsia	Anx (SAS), Dep (SDS)	(1) Intervention: psychosomatic care plus social media-based relaxation therapy (n=50), (2) control: routine nursing care (psychosomatic care) (n=50). Overall age (m= 47.6, sd=9.6)	(1) Psychosomatic care plus WeChat communication relaxation therapy: psychological counseling, emotional Zhi Nursing (traditional Chinese medicine), psychosomatic relaxation therapy, and physical exercise intervention. (2) Routine nursing care: psychological nursing, health education, diet, medication, and discharge guidance

[a] Asia (China, Hong Kong, India, Iran, Lebanon, Pakistan, Thailand, Vietnam), Africa (Kenya, Uganda, South Africa, Nigeria), Europe (Turkey, Romania), Latin America (Brazil, Colombia, Mexico)

[b]APAIS: Amsterdam Preoperative Anxiety and Information Scale; AVAT: Anxiety Visual Analog Test; BAI: Beck Anxiety Inventory; BDI-II: Beck Depression Inventory-II; CDI: Children's Depression Inventory; CES-D: Center for Epidemiological Studies-Depression; CFS: Children's Fear Scale; DASS-21: DASS: Depression Anxiety Stress Scale; DASS-21 is a short version of DASS; EPDS: Edinburgh Postpartum Depression Scale; EQ-5D-5L: measuring health related quality of life, depression and anxiety subscale; FIS: Facial Image Scale; FSAS: Florida Shock Anxiety Scale; GAD-7: General Anxiety Disorder scale-7; GAD-Q-IV: General Anxiety Disorder Questionnaire-IV; HADS: Hospital Anxiety and Depression Scale; HAMA: Hamilton Anxiety Rating; HAMD-17: Hamilton Depression Rating Scale; MOS-HIV: HIV-specific health related quality of life; PHQ-9: Patient Health Questionnaire-9; PHQ-8:

short version of PHQ-9; SAS: Self-Rating Anxiety Scale; SCAS: Spence Children's Anxiety Scale; SDS: Zung Self-Rating Depression Scale; SIAS: Social Interaction Anxiety Scale; STAI: Spielberger State Trait Anxiety Inventory; SQR-20: Self-Report Questionnaire-20; VAS: Visual Analog Scale; YPAS: Yale Preoperative Anxiety Scale; mYPAS: modified Yale Preoperative Anxiety Scale.
