# Supplementary material for: Effectiveness of Digital Mental Health Tools to Reduce Depressive and Anxiety Symptoms in Low- and Middle-Income Countries: Systematic Review and Meta-analysis
Source: JMIR Ment Health. 2023 Mar 20;10:e43066. doi: 10.2196/43066 (PMC10131603; doi:10.2196/43066)
Supplement: Multimedia Appendix 6 [file mental_v10i1e43066_app6.pdf]

Multimedia Appendix 6. Supplemental information of the studies for the systematic review

First author	Intervention duration	Inclusion criteria	Exclusion criteria
Abbasi 2021	1 day	Children of age 6 to 11 years who showed a willingness to take part in the study with no previous dental treatment or visit history and whose behavior could be rated as positive (+) or negative (-) based on "Wright's modification of the Frankl behavior rating scale."	Medically compromised children, those with disabilities, severe pain, facial swelling, and trauma were excluded from this study.
Adewuya 2019	14 weeks	Those scoring 10 and above on PHQ-9, intended to stay in the project area for at least 18 months, were literate enough to read either English, pidgin English or any of the three local languages (Yoruba, Hausa or Igbo), and completed the written informed consent form were enrolled in the trial.	Excluded were children (below 18 years), elderly (above 60 years), clients with serious medical condition or disability necessitating specialist care, having any form of psychosis, or under psychiatric care.
Ahorsu 2020	6 weeks	1) A diagnosis of epilepsy according to the International League Against Epilepsy criteria; 2) aged 18 years or older; 3) moderate or severe insomnia as indicated by a score of 15 or higher on the Insomnia Severity Index (ISI); 4) speak, understand, and write in Persian; 5) no surgery planned in the next 6 months; 6) access to an Android smartphone or a desktop computer with Internet access; and 7) absence of major cognitive impairment assessed using the Telephone version of the Mini-Mental State Examination.	1) Were diagnosed with a rapidly progressing neurological or medical disorder, 2) had an intellectual disability, 3) reported drug or alcohol misuse, 4) had untreated sleep apnea identified by an overnight (manually scored) polysomnography or 5) were pregnant.
Alessi 2021	16 weeks	Adults aged 18 years or older with a previous diagnosis of type 2 diabetes based on guideline recommendations; a HbA1c evaluation in the laboratory of the study's reference hospital in the three months prior to inclusion; and availability for weekly phone calls during the study.	Patients hospitalized at the time of recruitment and those who had some serious limitation preventing the necessary interaction, such as advanced dementia or severe hearing loss, were excluded.
Araya 2021	6 weeks	Adults (≥21 years) who reported receiving treatment for hypertension and/or diabetes at primary care units in São Paulo or attending ambulatory treatment. And those with ability to read a text on a smartphone screen were invited. Participants with clinically significant depressive symptoms (Patient Health Questionnaire-9 [PHQ-9] score 10) who were being treated for hypertension and/or diabetes.	Individuals assessed as having high suicide risk and pregnant women with gestational diabetes and/or hypertension at screening were excluded.
Arjadi 2018	10 weeks	1) Aged 16 years or older, scored 10 or above on the Patient Health Questionnaire 9 (PHQ-9), 2) met the criteria for major depressive disorder or persistent depressive disorder based on the Structured Clinical Interview for DSM-5, 3) were proficient in Bahasa Indonesia, and 4) could use the internet.	N/A
Asadzadeh 2020	4-6 weeks	1) 18 to 35 years old, 2) able to speak and read Persian (since some women were from the less privileged parts of Zanjan province in which all people do not speak Persian and did not have enough reading and speaking language skills), 3) in the last pregnancy trimester, and 4) having a single embryo.	1) Score ≥ 10 on the Edinburgh postnatal depression scale, 2) history of abortion and infertility, 3) mental or physical chronic diseases, 4) taking medicine that causes symptoms of depression, 5) history of postpartum depression in the first-degree relatives, and 6) experience of a major stressful event during the past year.
Baruah 2021	12 weeks	1) Age 18 years and older, 2) caregiver of the family member with Alzheimer's disease or dementia for at least 6 months, 3) residency in India, and 4) regular access to the Internet.	N/A
Byonanebye 2021	1 year	1) ART-naïve adults or ART-experienced people living with HIV, including key populations (sex workers and men who have sex with men), 2) young adults (18-24 years), 3) pregnant and breastfeeding mothers, 4) and people living with HIV in discordant relationships, 5) if they were 18 years or older, 6) were willing to comply with	1) People living with HIV with clinical conditions that could interfere with the use of cell phone (for example, deafness, severe cognitive impairment, critical illness), and 2) those who were not receiving the standard

		study procedures, and 7) had access to and were able to use a cell phone. 8) Participants also spoke English or one of the available local languages and provided informed consent.	first-line (efavirenz, tenofovir disoproxil fumarate, and lamivudine) or second-line (atazanavir or lopinavir with boosted ritonavir plus lamivudine and tenofovir) ART regimens.
Chan KL 2019	60 weeks	First-time expectant mothers receiving regular antenatal care services at KWH, were able to read and understand Chinese or English, and were willing to consent to the terms of the study.	People unable to give informed written consent or communicate with the interviewers. The participation of their partners was encouraged but was not a requirement for participation.
Chavooshi 2017	16 weeks	18–45 years of age, at least one MUP with a duration of six months, having access to a computer—desktop, laptop or tablet—and high-speed Internet at home (a minimum bandwidth of 2 Mbps was required), fluency in Farsi and provision of consent.	People with acute drug/alcohol abuse, psychotic or bipolar disorders, anorexia nervosa, acute suicidality or pregnancy.
Chavooshi 2016	16 weeks	18–45 years of age, at least 1 MUP with a duration of 6 months, having access to a computer—desktop, laptop, or tablet—and high-speed Internet at home (a minimum bandwidth of 2 Mbps was required), fluency in Farsi and provision of consent.	People with drug/alcohol abuse, psychotic or bipolar disorders, anorexia nervosa, acute suicidality, and pregnancy.
Chiang 2017	1 day	1) They were at least 18 years old and fit the definition of MFC, e.g., being the spouse, sibling, parent, and children, etc.; 2) their family members (patients) had stayed in ICU for at least 24 hours as identified by nurses; 3) they are able to read and write Chinese.	1) The patient was dying or had an APACHE II score of <15 or >24 (~25–40% mortality); and/or 2) the members of family caregiving had a known cognitive impairment or mental illness.
Ciuca 2018	12 weeks	1) Age within the range of 18–65 years, 2) having access to a computer with an internet connection, 3) being a native Romanian speaker, 4) exceeding the cutoff score on the PDSS-SR, 5) meeting the diagnostic criteria for panic disorder according to the diagnostic interview made over phone or Skype, 6) not being in another psychological treatment, 7) providing a second detailed informed consent for participating in the study.	Who exceeded a PDSS-SR cutoff score of at least six, individuals with comorbid severe psychiatric disorders such as bipolar disorders, psychotic disorders, and substance abuse or dependence. We also excluded participants with active suicidal plans according to clinical judgment.
Constant 2014	2-3 weeks	Scheduled to undergo a medical abortion at the clinic, over 18 years old, willing to comply with visit schedules, accessible by mobile phone, and comfortable with receiving abortion-related messaging following enrolment in the study.	N/A
Craveiro 2020	1 day	Needing primary endodontic treatment, being 18 years of age or older, signing a free and informed consent form, being no more active, and having filled out the proposed questionnaires completely.	Patients with systemic changes, who were pregnant, who had taken anxiolytics, antidepressants, or antihypertensives in the previous month, or who needed endodontic reintervention or surgery were excluded.
Cumino 2017	1 day	Unpremedicated, healthy children aged 4 to 8 years old inclusive, with American Society of Anesthesiologists (ASA) physical status I and II scheduled to undergo minor-to-moderate elective surgical procedures with general anesthesia.	Children with a history of developmental disabilities, neurological diseases or psychoactive medication use; children with hearing and/or visual impairment; children with a history of previous surgery.
Digin 2022	1 week	Patients who were adults, volunteered to participate in the study, accepted randomization, could communicate in Turkish, had a personal cell phone, could read text messages or were illiterate and had a relative to help them at home after discharge were included in the study.	Exclusion criteria included a known history of psychiatric disease or history of using psychiatric medication. In addition, patients who did not receive SMS reminder were excluded.
Duan 2018	8 weeks	Aged between 18 and 75 years, no restriction of physical mobility under the cardiac function at entry, no restriction of other relevant diseases such as diabetes or fruit allergies, sufficient reading, and writing skills in Chinese, internet access via a computer at home, and mobile access.	N/A
Duan 2017	8 weeks	Undergraduate students	Who were collegiate athletes, had restrictions in terms of PA or FVI, or because they declined to participate.

Duan 2022	8 weeks	1) Were not collegiate athletes or majoring in any sport-related subjects, 2) were not vegetarians, 3) had no restrictions on physical mobility (e.g., heart diseases, stroke, or disability) or FVC (e.g., fruit allergies or diabetes), and 4) were able to use a computer or laptop and mobile phone and had access to the internet.	N/A
Duruturk 2019	6 weeks	Ages between 18–65 years and diagnosis of type 2 DM at least 6 months.	Participants who are clinically unstable or who have a neuromuscular disease, unstable cardiovascular diseases, musculoskeletal disease, pregnancy, lactation, and inability or unwillingness to comply with the required exercise were excluded from the study.
Erdogan 2021	1 day	1) Being between the ages of 7 to 12 years, 2) being literate, and 3) requiring blood tests.	1) Having chronic diseases, 2) hospital stay for treatment, 3) visual, audio, or speech impairments, 4) mental disorders, 5) history of sedative, analgesic, or narcotic use within 24 h before admission, and 6) inflammatory disease during admission.
Gerceker 2016	1 week	Parents of children aged 3–17 years who had undergone outpatient surgery for appendicitis, cholecystectomy, and ovarian cysts were eligible to participate in the study.	Exclusion criteria were parents of children who had previous surgery or a chronic illness, parents with developmental, cognitive, language, or visual disabilities, and parents who were not the primary caregivers of the children.
Ghanbari 2021	4 weeks	The age range of 20 to 60 years, willingness to participate in the study, diagnosis of nonmetastatic breast cancer, literacy, access to smart mobile electronic devices connected to the internet and willingness to have the app installed on them, ability to work with the app and social networks, and moderate-to-severe anxiety (State-Trait Anxiety Inventory [STAI] score of >80) and low-to-moderate self-esteem (Rosenberg Self-Esteem Scale [RSES] score of <25).	The exclusion criteria consisted of failing to regularly participate in the educational or therapeutic program, serious diseases other than breast cancer, history of chronic psychological disorders and taking psychiatric drugs, and participation in similar psychoeducational programs, which could have biased the results.
Ghawadra 2020	4 weeks	Nurses who work in wards and who had mild to moderate levels of SAD (according to DASS-21) in an earlier cross-sectional survey.	Nurses who work in the outpatient clinic or nursing managers, due to the different types of patient care, roles, and responsibilities. Nurses who have a history of mental illness (n = 3) were excluded from the first study. The nurses who had severe and extremely severe levels of SAD (according to DASS-21). They were advised to seek professional help at the psychiatric/psychology clinic in the hospital.
Gu 2021	1 day	Patients having no visual/verbal/aural communication problems preventing the patient from understanding the information provided; between 18 and 45 years of age and subject to general anesthesia; and with an American Society of Anesthesiologists (ASA) physical status classification of grade I or II.	People not cooperating with the researcher; significant psychiatric disorders such as generalized anxiety disorder, panic disorder, depression, psychosis, and bipolar disorder; and individuals who were incompetent to give informed consent.
Guo L 2020	6 weeks	1) Aged 18 to 40 years; 2) in the second or third trimester of a pregnancy before 34 weeks; 3) present with antenatal depressive or anxiety symptoms defined by a score equal to or above 9 on the Edinburgh Postnatal Depression Scale (EPDS); and 4) have Internet access at home and proficient in reading and speaking Chinese.	1) Presence of serious physical condition related to pregnancy, including diabetes mellitus, severe high blood pressure, or eclampsia; 2) previous history or presence of psychiatric disorders including depression, psychosis, mania, and suicidal ideation, and on antipsychotics and psychotherapy currently or within the past 6 months; and 3) refuse to provide written consent.

Guo y 2020	12 weeks	1) Being 18 years or older, 2) being HIV seropositive, 3) having elevated depressive symptoms (measured by the Center for Epidemiologic Studies-Depression Scale [CES-D] ≥16), 4) willing to provide hair samples, and 5) using WeChat.	1) Currently on psychiatric treatment, 2) unable to finish the screening or baseline survey, 3) unable to read or listen to the materials sent via WeChat (e.g., short articles, audio, and posters), and 4) unable to engage in physical activities because of medical reasons.
Hamedi 2020	6 weeks	Patients with one type of cancer, age range from 9 to 12 years, literate and fluent in Persian.	Patients with a developmental psychiatric disorder, autism spectrum disorders (based on the diagnosis of a psychiatrist), and a history of seizure.
Hatipoglu 2018	1 day	American Society of Anesthesiologists physical Status I-II, aged 5–12 years old and scheduled for outpatient surgery (e.g., orchiopexy, hypospadias surgery, inguinal hernia, tonsillectomy, adenoidectomy, and strabismus surgery) were accepted in the present study.	Children with chronic illness, undergoing emergency surgery, cognitive disorders, and parents who refuse to participate were excluded from this study.
Heim 2021	8 weeks	Being able to understand and speak Arabic or English; access to an internet connected device; aged over 18; living in Lebanon; scores above the cutoff on the Patient Health Questionnaire and the WHO Disability Assessment Schedule 2.0.	N/A
Hua 2015	1 day	1) Aged 4-16 years, 2) with chronic wounds on the lower limbs that require active dressing changes.	Patients who are non-Chinese speaking, have a visual or auditory disability, have a diagnosed illness in addition to chronic wounds on the lower limbs, receive sedative medication, and have wounds that require surgery.
Huang 2018	N/A (During hospitalization)	Diagnosis in the third trimester meets the diagnostic criteria in Obstetrics and Gynecology, that is, pregnancy> 28 weeks; Chronic Hepatitis B diagnosis meets the criteria in Practical Internal Medicine; hepatitis B surface antigen positive> 6 months; serum HBV DNA> 10 5 /mL; persistent or intermittent alanine aminotransferase/aspartate aminotransferase level increased; liver biopsy revealed chronic hepatitis (inflammatory necrosis score ≥ 4 points). Inclusion criteria : 1) Those who meet the above diagnostic criteria; 2) Those who can use WeChat; 3) Those who have good compliance; 4) Those who have informed consent; 5) Those who have been approved by the hospital ethics committee.	1) Early and middle pregnancy; 2) Acute hepatitis B; 3) People with intellectual, hearing or mental impairment; 4) People with other liver diseases; 5) People with other infectious diseases; 6) Yes People with malignant tumors; 7) People with severe blood system diseases; 8) People who do not use WeChat or infrequently users; 9) People who have poor compliance.
Huang L 2021	12 weeks	18 years old or above; being first-time mothers with healthy babies; having the ability to respond the questionnaires; and being available to the internet by mobile phone or computer.	If they or their infants had serious diseases.
Imamura 2021	12 weeks	1) Currently employed full-time as a registered nurse, and 2) Can access the internet via a mobile device such as a smartphone.	1) Plan to change or quit the job in the next 7 months, 2) assistant nurses and helpers, 3) non-regular or part-time employed, 4) sick leave for 15 or more days for a physical or mental condition in the past 3 months, and 5) current treatment for a mental health problem from a mental health professional.
Inangil 2020	1 day	1) Being in the range of 7 to 12 years, 2) having a blood sample test ordered by the pediatric physician, 3) not having any acute pain or anxiety at the time of the procedure, 4) not having any audiovisual, cognitive sensitivity, or severe physical disability, and 5) having the ability to verbally communicate.	Incision or scar tissue in the forearm area, congenital, genetic, developmental, or neurologic disease, feeding or hydration problems, problems with skin integrity, or involuntary movement of the arm at the phlebotomy site.
Jannati 2020	8 weeks	Eligible study participants were women aged 18 or above. The inclusion criteria were having at least weekly access to the Internet and mobile phone and giving birth in the last six months and having sufficient Persian language skills to complete self-administered	N/A

		surveys. Moreover, the participants should get a score of 13 or higher on the Edinburgh Postnatal Depression Scale (EPDS).	
Jareethum 2008	12 weeks	Age over 18 years old, no medical diseases or obstetrics complications, singleton pregnancy, and dating confirmed by ultra-sound, gestational age less than 28 weeks when enrolled in the present study. All participants had their own mobile phone and could receive and understand SMS messages.	Pregnant women who aborted before28 weeks of gestation or changed to deliver at another hospital were excluded from the present study.
Khushnood 2021	8 weeks	Those included were vitally stable, physically independent with age 60 years and above, having no serious systemic disease.	Subjects with neurological conditions were excluded.
Korkmaz 2020	40 days	1) Underwent breast surgery (modified radical mastectomy or breast-conserving surgery) with axilla lymph node dissection, 2) did not have metastasis, 3) were at least 18 years old, 4) had an e-mail address, and 5) could use a computer or smartphone and had an internet connection.	1) Underwent breast cyst excision, 2) had arm and/or shoulder movement restrictions, 3) received breast cancer treatment, 4) received treatment for another type of cancer or were diagnosed with another type of cancer, or 5) had breast reconstruction.
Li 2021	4 weeks	1) Men who have had anal sex with at least one man in the last 6 months, 2) over 18 of age, 3) diagnosed as HIV-positive at least 3 months prior (as newly diagnosed PLWH tends to be unstable), 4) intending to stay in Chengdu for the coming 6 months, and 5) being a regular QQ user (i.e. using QQ at least once a week).	1) The presence of severe AIDS symptoms or other medical conditions, 2) having severe depression or suicidal ideation, 3) sharing their QQ account with others, and 4) utilizing psychiatric services or psychological counseling or participating in other interventions at the time of recruitment or during the study period.
Liu H 2022	16 weeks	Aged 18 years or older, being full-time university students, able to communicate in Chinese without difficulty, have skillful use of smartphones, have a PHQ-9 score of nine or higher, not currently undergoing any form of mental health intervention. The eligibility criterion of a PHQ-9 score equal to or greater than nine was decided according to the average inclusion PHQ-9 score in previous depression trials.	N/A
Liu Z 2021	1 week	Inclusion criteria those who were aged between 18-75 years (including threshold), regardless of gender; were under isolation observation while diagnosed with a mild or common type of COVID-19 and could cooperate to complete a corresponding psychological intervention; had mild to moderate depression or anxiety symptoms as defined by the 17-item Hamilton Depression Rating Scale (HAMD17) score≥7 or the Hamilton Anxiety Scale (HAMA) score≥7; had sufficient compliance to complete the experiment according to the protocol; and informed consent was provided by patients and (if necessary) guardians.	The exclusion criteria were patients who were clearly diagnosed with a psychiatric disorder, including depression, bipolar disorder, etc., in the 6 months prior to their diagnosis of COVID-19; patients with psychotic symptoms; patients with HAMD17 score≥24 or HAMA score≥21; patients with a high risk of suicide, defined as having a history of attempts by suicide in the 6 months prior to the study or who scored more than 3 on item three (suicide item) of the HAMD17 scale; patients with organic mental disorders; patients with substance abuse or dependence; patients undergoing treatment currently (pharmacological or psychological) for mental health problems; or patients presenting other conditions that the researchers believed were not suitable for this clinical trial.
Luo Y 2021	8 weeks	1) A child (aged 0-19 years) in whom cancer was diagnosed within the past year, 2) the ability to read Chinese and speak Mandarin, and 3) a smartphone with the WeChat app.	Parents with cognitive impairments or physical disabilities identified from medical records and those who were participating in other psychological interventions or consultations were excluded.
Luo YJ 2021	6 weeks	1) Interest and willingness to participate, 2) self-reported body dissatisfaction (“Are you currently dissatisfied with your body?”),	Recruitment was limited to women who reported body dissatisfaction because Body

		and 3) absence of DSM-IV diagnoses of anorexia nervosa, bulimia nervosa, or binge eating disorder based on responses to a validated eating disorder screen	Project interventions have typically produced larger effects when implemented selectively with high-risk groups relative to universal implementation.
Majd 2020	6 weeks	1) Being age 18 years or older, 2) having insomnia disorder according to the Diagnostic and Statistical Manual of Mental Disorders, Fifth Edition (DSM-5), 3) having an Insomnia Severity Index (ISI) score of 10 or higher, 4) understanding Persian, and 5) having access to a smartphone and/or desktop computer with internet access.	1) Had an uncontrolled medical condition that interfered with sleep or required immediate treatment (e.g., obstructive sleep apnea requiring continuous positive airway pressure treatment), 2) did shift work, 3) were pregnant, 4) were participating in other research and/or clinical trials, 5) had received psychotherapy in the past 6 months, 6) had current major depressive disorder based on the Structured Clinical Interview for DSM-5 disorders, 7) had a self-reported diagnosis of schizophrenia or psychosis, 8) showed evidence of alcohol abuse (more than 3 glasses of alcohol per day at least 21 days a month), 9) misused marijuana (use more than once per week), 10) appeared suicidal, or 11) had children aged under 2 years.
Mak 2015	8 weeks	All participants were above age 18 and were computer literate.	N/A
Mehri 2020	5 weeks	Parents with a child who had ADHD aged between 6–12 years, only using methylphenidate medications for the past 6 months, using fixed-dose of methylphenidate for last 30 days, having at least one disturbance in sleeping according to parents' reports based on the Children's Sleep Habits Questionnaire (CSHQ) (Owens, Spirito, & McGuinn, 2000), lacking another disease/disorder, having an Intelligence Quotient (IQ) of over 80, and living with at least one parent.	Parents who did not participate in training sessions even one session, discontinued participants' collaboration with researchers, variations in administration of the methylphenidate dose, and the start of other medications affecting ADHD and sleep status as well as any admission of the child to hospital during the study period.
Milani 2015	6 weeks	The criteria for including cases were term pregnancy, live birth and depression score (EPDS score) >10 to <14.	The criteria to exclude cases were: history of mental disorder, episodes of mental disorders during pregnancy or in the last 12 months that necessitated the use of medicines, nonviable fetus, history of PPD, current use of prescribed psychiatric drugs and an EPDS score of ≥14. Mothers with EPDS scores ≥14 and who had suicidal thoughts were referred to a psychiatrist. By considering the exclusion criteria, 21 of 75 depressed mothers were excluded; 54 of the participants were eligible to remain in the trial.
Moeini 2019	12 weeks	Female student, age 15-18 years, having access to the internet and the Center for Epidemiologic Studies Depression Scale (CES-D) score between 10 and 45 (mild and moderate depression).	Individuals with major depression were excluded from the study and were recommended to contact a psychiatrist. Other exclusion criteria were: do not access the computer or mobile phone for connecting to the internet, taking antidepressants, current participation in an intervention targeting depression, and moving to another school.
Mogoase 2017	1 week	Participants who showed stable dysphoria, operationalized as a score of at least 12 on the Beck Depression Inventory (BDI-II; Beck et al. 1996), at two consecutive assessments (i.e., initial screening and pre-training assessment).	N/A

Newman 2021	6 weeks	Eligible participants were aged 18 or older, provided their e-mail address, consented to participate, and met the criteria for clinical or subthreshold GAD. GAD status was measured by the Generalized Anxiety Disorder Questionnaire, the Fourth edition, which assesses the complete criteria for GAD as outlined in the Diagnostic and Statistical Manual of Mental Disorders, Fifth edition (DSM–5; American Psychiatric Association, 2013). Subthreshold GAD status was determined from scores above 5.7 on the GAD-Q-IV without fulfilling all GAD diagnostic criteria.	Participants were excluded if they were not interested in using the program, were receiving psychotherapy, or scored above 37 on the Posttraumatic Stress Disorder Checklist-5, indicating probable posttraumatic stress disorder.
Ngai 2015	5 weeks	Postpartum women who were 18 years of age or over, married, primiparous, Hong Kong residents, able to speak and read Chinese, giving birth to a single full-term healthy baby (gestation between 37 and 41 weeks; body weight >2.5 kg; APGAR score at 5 min >7) and scored ≥10 on the EPDS.	Women were excluded if they were single, had complications after delivery, had a regular psychiatric follow-up, or were currently taking antidepressant or antipsychotic drugs. An earlier study has demonstrated that the optimal cutoff score of the EPDS in a postnatal Chinese population is 9/10. Thus, women who score ≥10 on EPDS are regarded as having a high risk of postnatal depression.
Nobakht 2020	12 weeks	Mothers of children with Cerebral Palsy (CP) were included in this study if their children had Gross Motor Function Classification System (GMFCS) levels III, IV, and V, and aged between 4 to 12 years.	If the participants had two or more children with disabilities, or previously received face-to-face caring training, they were excluded from this study.
Ofoegbu 2020	10 weeks	Having depression, being an educational technology student of a Nigerian Federal University, not being currently in any depression intervention or psychotherapy, having a laptop, iPhone, tablet, or smartphone that accesses the internet, and being willing to sign informed consent to participate in the study.	Having the presence of bipolar or psychotic subtypes of depression, panic disorder, current substance abuse, past or present schizophrenia, organic brain syndrome, being currently in educational intervention, psychotherapy, psychotropic medication or hospitalization for psychosis, or risk of imminent suicide, as in the previous study.
Osborn 2020	1 day	Students aged13–18 were eligible. No exclusion criteria were applied.	N/A
Pakrad 2021	12 weeks	The study population comprised patients having Coronary Artery Bypass Surgery (CABG) at the center.	1) New York Heart Association class III or IV, 2) severe musculoskeletal issues, 3) positive exercise test (e.g., ischemia), 4) no smartphone, and 5) serious mental illness (indicated by taking medication for psychiatric disorders).
Peng 2018	8 weeks	A primary diagnosis of chronic Heart Failure (HF) for at least 3 months; New York Heart Association (NYHA) classification I to III; more than 18 years of age; a clinically stable condition with a regular medication regimen for at least 4 weeks before enrolment in the study; the ability to use WeChat or QQ software via a smartphone; discharged to home; and the ability to understand and speak Chinese.	The exclusion criteria for the patients included: myocardial infarction within the last month, unstable angina, uncontrolled hypertension, severe respiratory diseases, decompensated non-cardiac disease, malignancy, physical disability, mental disease, or other contraindications that affected participation in this study surgical treatment within the last month; and previous participation in exercise cardiac rehabilitation programs.
Rad 2018	2 weeks	N/A	N/A
Rahimi 2021	6 weeks	Women with failed IVF cycles, minimum educational attainment of junior high school, living in Tabriz, having a landline telephone number and a mobile phone number and getting a score of 8 or higher on the anxiety questions of the DASS-21 scale.	The history of psychiatric problems, self-reported psychotropic medication use, self-reported addiction to drugs, cigarettes, and alcohol, self-reported history of chronic physical problems (cardiac disorders, hypertension, pulmonary diseases, iron

			deficiency anemia, diabetes, thyroid disorders, epilepsy), and self-reported severe psychological crisis during the last 3 months, such as the death of relatives.
Salamanca-Sanabria 2020	7 weeks	Age ≥18 years. Mild to moderately severe depressive symptoms determined by the Patient Health Questionnaire - 9 scores of 10-19.	Severe depressive symptoms: score of >19 on the Patient Health Questionnaire - 9. Suicidal ideation or intent: score of ≥2 on question 9 of the Patient Health Questionnaire - 9 Psychosis. Currently in psychological treatment for depression. On medication for <1 month, alcohol or drug misuse, previous diagnosis of an organic mental health disorder, depression preceding or coinciding with a diagnosed medical condition.
Shahdosti 2020	2 days	Willingness to participate in the study, the age range of 18 -75 years, being in a conscious state, ability to verbally communicate, lack of a history of known anxiety and depression, undergoing coronary artery bypass graft (CABG) for the first time, and a smartphone with an internet connection to be used by patients' families.	Hemodynamic instability, decreased consciousness, returning to the operating room for any reason, need for reintubation, and inability to communicate through video calls more than two times.
Sivrikaya 2021	1 day	Who underwent impacted lower right third molar extraction.	Patients who previously obtained information from social media, had a bad dental treatment experience, had a serious systemic disease, or were pregnant were not included in the study.
Song 2021	12 weeks	Fulfilling the modified New York classification criteria for ankylosing spondylitis (AS), aged ≥ 14 years, able to understand/read Chinese, able to use WeChat, and willing to participate in this study.	Who had severe psychological and cognitive impairment, and were participating in other studies. Patients with other rheumatic diseases were also excluded.
Srivastava 2020	12 weeks	Both male and female adolescents within an age range of 13−19 years having the ICD-10 diagnosis of Mild/ Moderate Unipolar Depression (World Health Organization, 1992) with average IQ, working knowledge of computers and English were included in the study. Participants were included in the study if their medication was stabilized for 4 weeks.	Participants were excluded if they had active suicidal ideation or they met a current diagnosis of bipolar affective disorder, anxiety disorders, dissociative disorders, ADHD, learning disability, conduct disorder, oppositional disorder, psychoactive substance abuse or dependence; had a history of head injury, epilepsy, history suggestive of organic disorder or had received formal psychological intervention for depression in the past and had any major disability that might limit them from using computers.
Stamm 2018	15 days	Patients undergoing radiotherapy, aged 18 years or older were included; under radiotherapy treatment due to a diagnosis of cancer; and who had a telephone.	Being in no clinical, cognitive and/or communicative conditions
Su 2021	12 weeks	Adults who had been hospitalized for an initial diagnosis of Coronary Heart Disease (CHD) based on angiography or the exacerbation of CHD in previously diagnosed cases, were discharged, could read and speak Chinese, had an education background at the primary level or above, were able to use a computer and/or smartphone to access the internet and had no prescribed physical activity restrictions.	Patients with a life-limiting condition, acute psychotic disease, absolute or relative contraindications to exercise testing and training, and a high risk of exercise prescription based on the American Association of Cardiovascular and Pulmonary Rehabilitation guidelines or visual, fine motor, auditory or ambulatory disorders were excluded.
Taleban 2016	4 weeks	Ages were over 18 years old, had accessibility to cell phones, and met our inclusion criteria. Not met criteria consisted of mental retardation (MR), visual disability leading to reading impediments,	Exclusion criteria were a patient disinclination to continue our treatment strategy.

		dementia, suicide attempt history, electrotherapy, and receiving psychological intervention, as well as drug abuse and alcohol addiction.	
Tam 2020	6 weeks	These two schools were in areas sharing similar characteristics, including 1) areas mainly accommodating rural-to-urban migrant families, and 2) areas situated in communities with low social educational status.	N/A
Thitipitchayanant 2018	4 weeks	Willing and nulliparous mothers who were screened by Stein's postpartum blues questionnaire ≥ 3 and screened by Edinburgh Perinatal Depression Scale (EPDS) < 13; maternal aged 20 to 35 years.	Those who had complications from medical and obstetrical complication; psychosis disorder; on antipsychotic medication, unable to understand and read Thai; and unaltered accommodation after three months of childbirth were excluded.
Tiburcio 2018	8 weeks	1) Aged 17 years of age or older, 2) low-to-moderate drug-related risk, and 3) negative report of suicidal ideation in the previous 3 months.	N/A
Tol 2020	1 week	The independent assessors administered the Kessler 6 (K6) to assess psychological distress, applying a cutoff score of five or more for moderate-level psychological distress.	Participants were excluded if they were at imminent risk of suicide (assessed with a structured questionnaire); showed observable signs of severe mental disorder (e.g., psychosis); or were not able to understand basic instructions, with the latter two assessed with observation checklists.
Torabizadeh 2021	1 week	1) The scheduled coronary artery angiography would be the first experience of the patient with the procedure; 2) an age range of 25–75 years (the common age range in adults for coronary angiography); 3) lack of a job related to medical care and treatment; 4) ability to receive text messages via cell phone and to use a computer for watching multimedia content stored on a DVD; 5) literacy (defined as being able to read and write with completion of a minimum of five years of elementary school); and 6) a minimum of four days being left until the angiography appointment.	1) Experience of another invasive diagnostic procedure, such as transesophageal echocardiography or prior angiography; 2) a low level of consciousness or known case of a psychological disorder; and 3) a positive history of psychiatric medication use.
Tulbure 2018	9 weeks	1) Fluent in Romanian, 2) at least 18 years of age, 3) a BDI-II score between 14 and 50, 4) a diagnosis of current major depression disorder (MDD) or dysthymia using the Structural Clinical Interview for DSM-IV (SCID-I), 5) no suicidal ideation, 6) either not taking antidepressant medication or taking a stable dose during the past month, 7) not currently in a psychosocial treatment program for depression, 8) having Internet access, and 9) not taking medication for bipolar disorder or psychosis (i.e., used as a proxy for these diagnoses).	N/A
Tulbure 2015	9 weeks	1) Being over 18 years old, 2) exceeding the cutoff score on Social Phobia Inventory (SPIN) (i.e., SPIN total score ≥ 19), Social Interaction and Anxiety Scale (SIAS) (i.e., SIAS total score ≥ 24), and Liebowitz Social Anxiety Scale—Self Report version (LSAS-SR) (i.e., LSAS-SR total score ≥ 30), 3) fulfilling the DSM-IV criteria for SAD on Social Phobia Screening Questionnaire (SPSQ), 4) having SAD as the primary diagnostic on Structured Clinical Interview for DSM-IV-TR (SCID), 5) presenting no suicidal ideation (i.e., not exceeding a score of 2 on the suicide item of Beck Depression Inventory-II (BDI-II), and not reporting parasuicidal behavior on the Screening Questionnaire of the SCID), 6) not currently receiving other forms of psychological treatment for SAD, 7) having access to a computer connected to the internet, 8) if on medication, the dose should be constant for at least 1 month, and	N/A

		participants should agree to keep the dosage unchanged for the whole duration of the study, and 9) having no diagnosis of psychoses or borderline personality disorder on the SCID.	
Wang 2020	8 weeks	Participants who were older than 18 years and who met the diagnostic criteria of SAD in the Structural Clinical Interview for Diagnostic and Statistical Manual of Mental Disorders, 4th edition (DSM-IV) Axis I Disorders. Their Social Interaction Anxiety Scale (SIAS) score was higher than 22, with Social Phobia Scale (SPS) score higher than 33. They did not take any antipsychotic drugs or undergo other psychological treatments in the last year, and they did not meet the diagnostic criteria of schizophrenia, bipolar disorder, and high suicidal tendency. Participants had to agree that they could finish the 8-week ICBT program and the posttreatment measurements.	N/A
Wantanakorn 2018	1 day	Children age 5 to 12 years old who need bone marrow aspiration procedures.	N/A
Wei 2020	2 weeks	1) Aged 18–65 years; 2) PHQ-9 or GAD-7 of ≥5; 3) completed at least a junior middle school level of education.	Any participant who met one of the following criteria was excluded: 1) PHQ-9 or GAD-7 of ≥15; 2) with suicidal ideation; 3) use of any antipsychotics; 4) the underlying disease was too severe to complete the assessments; 5) unable to follow the instructions of the internet-based intervention.
Xia 2020	12 weeks	Inclusion criteria included 1) the subject must have a permanent colostomy after colorectal surgery; 2) subjects must be between the ages of 18 and 70 and agree to participate and fill out the questionnaire; 3) subjects must be able to read and write and understand the questionnaire; and 4) subjects must be willing to have a return visit in which they participate in a teaching plan about the care of their colostomy.	Exclusion criteria were as follows: 1) serious cardiovascular, endocrine, or other serious diseases (other than the diagnosis of colorectal cancer). 2) Presence of emotional or cognitive disorders such that the patient was incapable of fulfilling the requirements of the study. The study was approved by the ethics committee of the First Affiliated Hospital of Wenzhou Medical University, and written informed consent was obtained from all participants.
Yan 2022	4 weeks	The inclusion criteria were as follows: 1) premature infants; 2) parents were the primary caregivers; 3) parents can use WeChat correctly; and 4) the Internet was easily accessible at home.	The exclusion criteria were as follows: 1) complications of serious diseases, such as congenital heart disease and digestive tract malformation; and 2) parents declined to participate in the study.
Yang B 2021	12 weeks	1) Premature infants with Patent Ductus Arteriosus (PDA); 2) parents were the primary caregivers; and 3) parents had smartphones and were proficient in using the WeChat platform.	1) Patients complicated with other congenital heart malformations; 2) severe conditions requiring an emergency operation or long-term drug treatment; 3) patients complicated with other neonatal diseases; and 4) parents refusing to participate in the study or follow-up program.
Yang L 2019	1 year	1) Diagnosed with coronary artery disease, which was defined as at least 1 coronary artery having stenosis greater than the cutoff point of 50% by coronary angiography; 2) older than 18 years; 3) having depression, which was defined as a HADS-D score greater than the cutoff point of 8 and an SDS score greater than the cutoff point of 50; 4) a life expectancy of more than 1 year, which was evaluated by an experienced specialist according to the patient's conditions, such as disease severity, medical history, and complications, and 5) available to be followed up regularly, which was assessed by the	1) Treated with antidepressants within 3 months before enrollment; 2) history of other mental disorders, such as dementia, schizophrenia, schizotypal affective disorder, delusional disorder, bipolar affective disorder, alienation, schizoid personality disorder, and others; 3) imminent risk of suicide, which was assessed by the investigator according to the patient's psychologic status, severity of depression, and history of suicide attempts; 4)

		investigator according to the patient's disease condition, financial situation, home location, and willingness.	uncontrolled hypertension, cardiac arrhythmia, or unstable angina pectoris; 5) history of severe pulmonary and renal comorbidities, heart failure, tumors, or other life-threatening diseases; or 6) pregnancy or lactation.
Yang M 2019	8 weeks	The inclusion criteria were women aged more than 18 years, 24 to 30 weeks' gestation, low-risk pregnancy at the start of the intervention, internet access, fluent in Chinese and able to complete the questionnaires, and elevated depressive or anxious symptoms as determined by either a PHQ-9 score of more than 4 or a GAD-7 score of more than 4.	The exclusion criteria were history or current diagnosis of a psychosomatic disease (physical symptoms or illness that results from the interplay of psychosocial and physiologic processes, such as hypertension, diabetes mellitus, or asthma), current substance abuse, previous participation in psychological therapy or a stress reduction program, history of suicide attempts, current use of any psychoactive drug, and a high level of depression (PHQ-9 score >14) or anxiety (GAD-7 score >14). Women who had regular mind-body practice (yoga, meditation, or mindfulness practice) were also excluded from the study.
Yardimci 2019	72 weeks	The inclusion criteria were voluntarily agreeing to participate in the study, having an Implantable Cardioverter Defibrillator (ICD), being able to use a computer and the Internet, being able to understand and speak Turkish, and age >18 years.	Patients diagnosed with neurologic disorders (such as Alzheimer's) and psychiatric disorders (such as schizophrenia) were excluded from the study.
Yeung 2018	5 weeks	The inclusion criteria for the study were: 1) Self-identification as Chinese origin; 2) At least 18 years of age; 3) Proficiency in Chinese, including the ability to read Chinese; 4) Access to a computer and the internet; 5) Significant depressive symptoms as judged by the patient's treating clinicians.	The exclusion criteria were: 1) Use of illicit drugs or consumption of more than three standard drinks in a day. 2) Current Symptoms of psychosis. 3) Past or current history of schizophrenia or bipolar disorder. 4) Electroconvulsive treatment in the past year. 5) Active suicidality or self-injurious potential necessitating immediate intervention.
Zengin 2021	4 weeks	Parents with children aged 3-6 years who attended a daycare center or nursery during the pre-pandemic period, who had had a pre-test anxiety scale score above 40, a computer or internet infrastructure at home and volunteered to participate in the study were included in the research.	Parents who had communication problems, had a pre-test anxiety scale score below 40, did not volunteered to participate in the study, used drugs for anxiety or depression, did not have a computer or internet environment, did not attend at least one session were excluded from the study.
Zhang QL 2021	4 weeks	The inclusion criteria were as follows: 1) infants that had undergone Congenital Heart Disease (CHD) surgery; 2) the infants' parents were the primary caregivers; 3) the parents had smartphones and could use WeChat properly and 4) the internet was easy to use at home.	The exclusion criteria were as follows: 1) infants that had other serious diseases; 2) infants that experienced postoperative death and 3) the parents of the infants that refused to participate in the study.
Zhang QL 2021	1 day	The inclusion criteria were as follows: 1) children with restrictive ventricular septal defects; 2) surgical treatment after the age of 1 year; 3) parents as the main care givers; and 4) using WeChat correctly.	The exclusion criteria were as follows: 2) moderate to severe pulmonary hypertension; 2) requiring surgery within 1 year of age; 3) other congenital heart diseases; and 4) parents' refusal to participate in the study or follow-up plan.
Zhang X 2021	4 weeks	The inclusion criteria were as follows: 1) ≥18-year-old women; 2) Edinburgh Postnatal Depression Scale (EPDS) scores ≥9 or Generalized Anxiety Disorder 7-item Scale (GAD-7) scores ≥5; 3) single pregnancy; 4) at 12–24 gestational weeks; 5) at least junior	Exclusion criteria were as follows: 1) the presence of suicidal ideation (score ≥1 on item 10 of the EPDS); 2) severe mental disorders (e.g., a diagnosis of psychotic disorders) or physical diseases (e.g., cancer or cardio-

		middle school educational level; 6) fluent in Chinese; and 7) not part of or on the waiting list of another psychological intervention.	cerebrovascular diseases); and 3) prior mindfulness experience.
Zhang Y 2021	6 weeks	Inclusion criteria were 1) aged between 18 and 30 years, 2) able to read and write in Chinese, 3) willing and able to perform moderate-to-vigorous physical activity (MVPA), 4) without self-reported cancer diagnosis or history of psychosis, hypertension, or heart disease that would affect their involvement in PA, 5) without regular exercise habits and failure to meet the current international physical activity recommendation (i.e., at least 30 min of MVPA for at least 5 days per week) in the past year, 6) self-reported characteristics of sedentary and anxious/stressed, and 7) intention to improve their quality of life by gaining health-related knowledge or increasing their MVPA.	N/A
Zhao 2021	10 weeks	The inclusion criteria for selecting participants were as follows: 1) availability of one parent and one child, 2) children's age was between three and ten years old, and 3) children were diagnosed with ASD based on DSM-V criteria.	N/A
Zheng 2021	2 weeks	Inclusion criteria were as follows: 1) grade 7 (12-13 years old) students in Duanzhou district and 2) under home confinement and enrolled in online learning courses during the COVID-19 outbreak.	Exclusion criteria included the presence of disorders such as autism, pervasive developmental delay, and schizophrenia, which might interfere with participation in the intervention. The study followed the CONSORT (Consolidated Standards of Reporting Trials) guideline. The reporting of the mobile-phone–based questionnaires followed the CHERRIES checklist (Checklist for Reporting Results of Internet E-Surveys).
Zhianfar 2020	4 weeks	Inclusion criteria were being diagnosed with the ESRD and receiving hemodialysis for at least three months, being 18 years of age or older and being independent for doing daily activities (walking, eating, etc.) with at least a reading and writing literacy level.	The exclusion criteria were having a diagnosis of mental or cognitive problems, use of antidepressants and having a depression score of less than four (according to the Beck Depression Inventory scoring mechanism).
Zhou 2019	4 weeks	Participants were women with breast cancer admitted to hospital, aged 18 years and above, and newly diagnosed with breast cancer. All patients were preparing to receive surgery and other adjuvant therapy.	Exclusion criteria were the presence of cognitive and psychiatric disorders (as screened and diagnosed by a psychiatrist not involved in this study and according to the Diagnostic and Statistical Manual of Mental Disorders, 5th ed.), other malignant tumors, and breast disease.
Zhuang 2017	2 weeks	Inclusion criteria: 1) Those who meet the above diagnostic criteria; 2) The course of the disease is at least 6 months; 3) The age is 30-68 years old; 4) All patients signed the informed consent; 5) can use WeChat; 6) follow the researcher.	Exclusion criteria: 1) Those with organic diseases; 2) Severe heart, brain, liver, kidney and hematopoietic system diseases; 3) Malignant tumors in the gastrointestinal tract 4) Pregnant or breastfeeding women; 5) Those with hearing, intellectual and mental disabilities; 6) Those who do not comply with this researcher.
