# Supplementary material for: Effectiveness of Digital Mental Health Tools to Reduce Depressive and Anxiety Symptoms in Low- and Middle-Income Countries: Systematic Review and Meta-analysis
Source: JMIR Ment Health. 2023 Mar 20;10:e43066. doi: 10.2196/43066 (PMC10131603; doi:10.2196/43066)
Supplement: Multimedia Appendix 7 [file mental_v10i1e43066_app7.pdf]

Multimedia Appendix 7. Supplemental information of the sensitivity analysis

1) Pre-planned sensitivity analysis

- When poor quality studies were excluded;

  When the weak-rated studies were excluded (n=13 studies), the overall effect size slightly decreased for depression from Hedges' g = -0.61 (95% CI= -0.78, -0.44) to Hedges' g = -0.59 (95% CI= -0.79, -0.40)

  When the weak-rated studies were excluded (n=10 studies), the overall effect size increased for anxiety from Hedges' g = -0.73 (95% CI= -0.93, -0.53) to Hedges' g = -0.77 (95% CI= -1.01, -0.52)

- When the psychometric scores were replaced with the other scores for the studies reported more than one measurement for depression and anxiety outcomes;

  When APAIS was replaced with AVAT for the study Gu et al 2021, the overall effect size for anxiety stayed the same, Hedges' g = -0.73 (95% CI= -0.93, -0.53).

  When SAS was replaced with HAMA, STAI-T, STAI-S, VAS for the study Liu Z et al 2021, the overall effect sizes for anxiety slightly changed like below;

  Hedges' g = -0.73 (95% CI= -0.93, -0.53) [HAMA]
  Hedges' g = -0.73 (95% CI= -0.93, -0.52) [STAI-T]
  Hedges' g = -0.72 (95% CI= -0.93, -0.51) [STAI-S]
  Hedges' g = -0.73 (95% CI= -0.94, -0.53) [VAS]

- When the studies excluded that the difference of pre-intervention psychometric scores between intervention and control groups exceeded the difference of post-intervention scores (n=8 for depression and n=6 for anxiety);

  the overall effect size greatly increased both for depression from Hedges' g = -0.61 (95% CI= -0.78, -0.44) to Hedges' g = -0.70 (95% CI= -0.90, -0.51) and anxiety from Hedges' g = -0.73 (95% CI= -0.93, -0.53) to Hedges' g = -0.85 (95% CI= -1.08, -0.62)

- When the studies were excluded that the digital mental heal tool was an adjunct to the non-digital main intervention (n=1 for depression and n=3 for anxiety);

  the overall effect size increased both for depression from Hedges' g = -0.61 (95% CI= -0.78, -0.44) to Hedges' g = -0.62 (95% CI= -0.79, -0.45) and for anxiety from Hedges' g = -0.73 (95% CI= -0.93, -0.53) to Hedges' g = -0.74 (95% CI= -0.95, -0.52)

2) Post-hoc sensitivity analysis for the outliers (n=22 for depression and n=19 for anxiety).

  When the outliers were excluded, the overall effect sizes decreased both for depression from Hedges' g = -0.61 (95% CI= -0.78, -0.44) to Hedges' g = -0.57 (95% CI= -0.66, -0.48) and for anxiety from Hedges' g = -0.73 (95% CI= -0.93, -0.53) to Hedges' g = -0.66 (95% CI= -0.77, -0.56).
