# Supplementary material for: Effectiveness of Digital Mental Health Tools to Reduce Depressive and Anxiety Symptoms in Low- and Middle-Income Countries: Systematic Review and Meta-analysis
Source: JMIR Ment Health. 2023 Mar 20;10:e43066. doi: 10.2196/43066 (PMC10131603; doi:10.2196/43066)
Supplement: Multimedia Appendix 8 [file mental_v10i1e43066_app8.pdf]

Multimedia Appendix 8. Funnel plots for depression and anxiety

1) Depression

a) Funnel plot

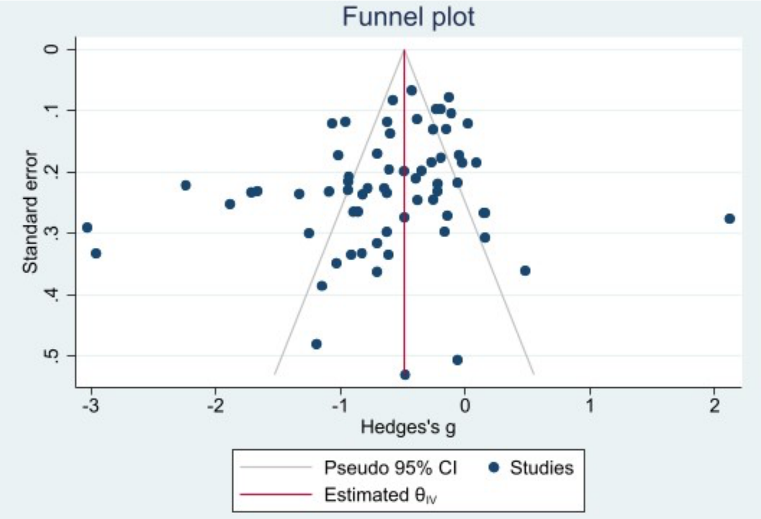

b) Egger's test result: β = -1.24, SE=0.95, p-value=0.19

c) Duval and Tweedie's trim and filled test result:

Studies	Hedges' g	95% CI
Observed	-0.61	-0.78 to -0.44
Observed + Imputed	-0.61	-0.78 to -0.44

2) Anxiety

a) Funnel plot

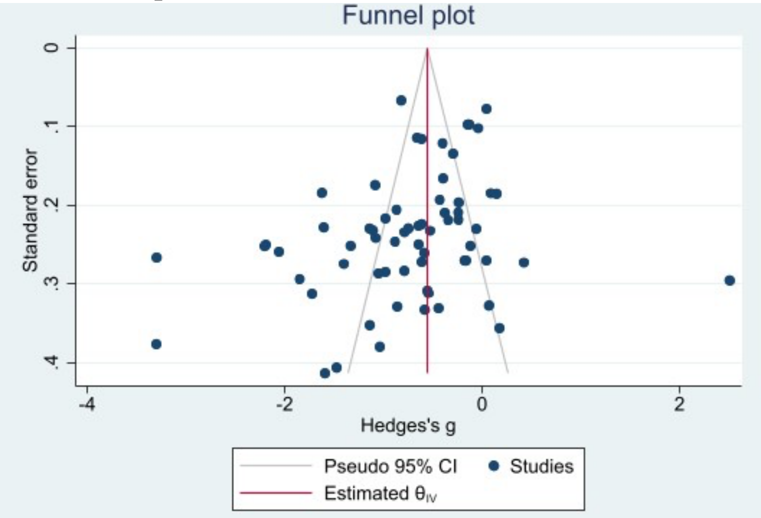

b) Egger's test result: β = -2.78, SE= 1.30, p-value=0.03

c) Duval and Tweedie's trim and filled test result:

Studies	Hedges' g	95% CI
Observed	-0.73	-0.93 to -0.53
Observed + Imputed	-0.73	-0.93 to -0.53
